# Nur77-activated lncRNA WFDC21P attenuates hepatocarcinogenesis via modulating glycolysis

**DOI:** 10.1038/s41388-020-1158-y

**Published:** 2020-01-20

**Authors:** Yun-feng Guan, Qiao-ling Huang, Yuan-li Ai, Qi-tao Chen, Wen-xiu Zhao, Xiao-min Wang, Qiao Wu, Hang-zi Chen

**Affiliations:** 1 State Key Laboratory of Cellular Stress Biology, Innovation Center for Cell Signaling Network, School of Life Sciences, Xiamen University, Xiamen, 361102 Fujian PR China; 2 Fujian Provincial Key Laboratory of Chronic Liver Disease and Hepatocellular Carcinoma, Zhong Shan Hospital, Xiamen University, Xiamen, 361005 Fujian PR China

**Keywords:** Cancer metabolism, Nuclear receptors, Non-coding RNAs

## Abstract

Hepatocellular carcinoma (HCC) is one of the leading causes of cancer-related mortality worldwide. Orphan nuclear receptor Nur77, which is low expressed in HCC, functions as a tumor suppressor to suppress HCC. However, the detailed mechanism is still not well understood. Here, we demonstrate that Nur77 could inhibit HCC development via transcriptional activation of the lncRNA WAP four-disulfide core domain 21 pseudogene (WFDC21P). Nur77 binds to its response elements on the WFDC21P promoter to directly induce WFDC21P transcription, which inhibits HCC cell proliferation, tumor growth, and tumor metastasis both in vitro and in vivo. In clinical HCC samples, WFDC21P expression positively correlated with that of Nur77, and the loss of WFDC21P is associated with worse prognosis. Mechanistically, WFDC21P could inhibit glycolysis by simultaneously interacting with PFKP and PKM2, two key enzymes in glycolysis. These interactions not only abrogate the tetramer formation of PFKP to impede its catalytic activity but also prevent the nuclear translocation of PKM2 to suppress its function as a transcriptional coactivator. Cytosporone-B (Csn-B), an agonist for Nur77, could stimulate WFDC21P expression and suppress HCC in a WFDC21P-dependent manner. Therefore, our study reveals a new HCC suppressor and connects the glycolytic remodeling of HCC with the Nur77-WFDC21P-PFKP/PKM2 axis.

## Introduction

Liver cancer is one of the most common types of cancer and, after lung cancer, is the second leading cause of cancer-related mortality worldwide [1]. Hepatocellular carcinoma (HCC), the main histological subtype of liver cancer, leads to more than 700,000 deaths per year globally [1, 2]. Although recent studies have shown that hepatitis B virus or hepatitis C virus infections and nonalcoholic steatohepatitis are the major risk factors for HCC [3], many aspects, especially the mechanisms underlying metabolic regulation in hepatocellular tumorigenesis and progression, are still largely unknown.

Long noncoding RNAs (lncRNAs) are transcripts longer than 200 nucleotides (nt) with no apparent protein coding potential [4, 5]. Mounting evidence has shown that lncRNAs are frequently deregulated in liver cancer and play important roles in the hepatocarcinogenic process [6, 7]. Therefore, lncRNAs are considered suitable biomarkers and therapeutic targets for HCC [6]. LncRNAs mainly exert their functions by interacting with DNA, RNA, or proteins to regulate signal transduction pathways or metabolic modes in cells [8, 9]. For example, the lncRNA HOX transcript antisense RNA, which is highly expressed in HCC samples and correlates with hepatocarcinogenesis and metastasis [10], functions as not only a modular scaffold that regulates histone modification and protein ubiquitination through interactions with proteins but also a molecular miRNA sponge to suppress the functions of miRNA-218 [10–12]. At least 75% of the mammalian genome is actively transcribed into noncoding RNAs, the majority of which are lncRNAs [13]; therefore, further understanding the functions and regulatory mechanisms of lncRNAs in HCC is of great practical importance.

Orphan nuclear receptor Nur77, also known as TR3 or NGFI-B, is encoded by the immediate early gene *Nr4a1* and plays paradoxical roles in the development of many cancers, including HCC [14–17]. As a transcriptional factor, Nur77 could exert its biological functions through regulating the expression of its downstream targets [18]. For example, upon stimulation with the chemotherapy drug cisplatin, Nur77 transcriptionally inhibits the expression of the anti-apoptotic genes BRE and RNF-7, thereby promoting cisplatin-induced tumor cell apoptosis [19]. On the other hand, the nongenomic activities of Nur77 are also vital for Nur77-mediated regulation [20]. Recently, our study demonstrated that Nur77 interacts with and stabilizes PEPCK1, the rate-limiting enzyme in gluconeogenesis, by impeding the SUMOylation and ubiquitination of PEPCK1, thereby facilitating gluconeogenesis in HCC cells and suppressing HCC progression [21]. However, whether the transcriptional regulation activity of Nur77 is also involved in HCC inhibition remains to be elucidated.

In this study, we found that Nur77 transcriptionally induces the expression of the lncRNA WFDC21P in HCC cells, which inhibits HCC cell proliferation and metastasis both in vitro and in vivo. In clinical samples, WFDC21P is low expressed in HCC samples than in paracarcinoma tissues, and the expression of WFDC21P positively correlated with the prognosis of HCC patients. Mechanistic analysis reveals that the inhibitory effect of WFDC21P in HCC is closely linked with the modulation of glycolysis via interacting with PFKP and PKM2.

## Results

### Nur77 transcriptionally upregulates lncRNA–WFDC21P expression in HCC cells

**Fig. 1:**
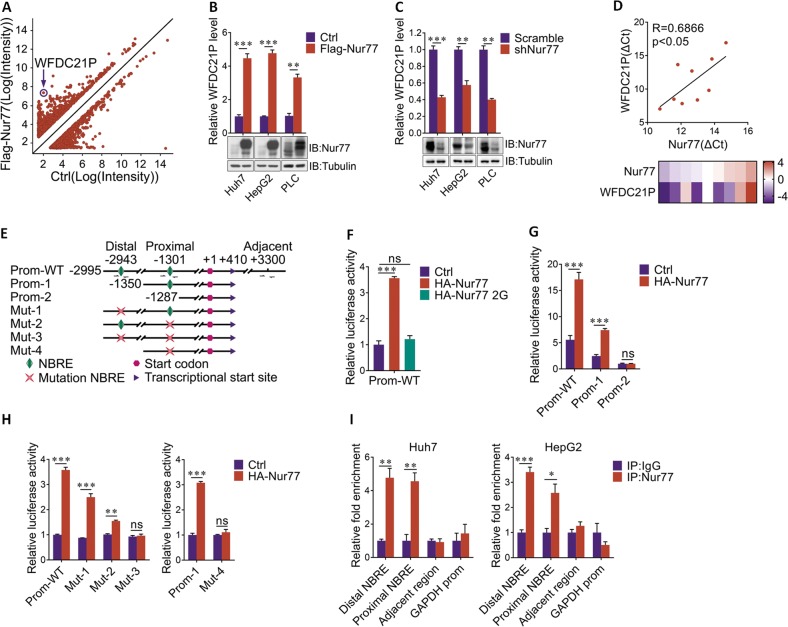
Nur77 transcriptional activates the expression of lncRNA–WFDC21P. **a** The scatter plot analysis of the lncRNA microarray data. LncRNAs that were differentially expressed (fold-change > 1.5) between control Huh7 cells and Huh7 cells overexpressing Nur77 are shown. **b**, **c** Nur77 promotes WFDC21P expression. Nur77 was overexpressed (**b**) or knocked down (**c**) in Huh7, HepG2, and PLC cells. WFDC21P expression levels were determined by RT-qPCR, and the protein levels of Nur77 were determined by western blotting. **d** Heat maps and correlation charts show the positive correlation between Nur77 and WFDC21P in L02 hepatocyte and eight HCC cell lines (Huh7, HepG2, BEL7402, SK-HEP-1, MHCC-97H, Hep3B, BEL7404, and MHCC-97L). The ΔCt values (normalized to β-actin) were subjected to Pearson’s correlation analysis or heat map diagraming. **e** Schematic diagram of the luciferase reporters of the WFDC21P promoter. **f** Nur77 enhances the activity of the WFDC21P promoter. A luciferase assay was performed in Huh7 cells. **g**, **h** Nur77 activates the transcriptional activity of the WFDC21P promoter in a NBRE-dependent manner, revealed by a luciferase assay in Huh7 cells. **i** Nur77 binds to the promoter of WFDC21P. ChIP assays were performed in Huh7 and HepG2 cells. The primers for the ChIP assays are indicated with arrows in **e**. GAPDH promoter amplification is used as a negative control. The data are represented as the means ± SEM of at least three independent experiments. *p < 0.05; **p < 0.01; ***p < 0.001 and ns no significance.

Our previous studies have shown that Nur77 could suppress HCC independent on its transcriptional activity [21]. Here, we further found that although Nur77 2G (a Nur77 mutant that lost its DNA binding ability due to 2 Cys to Gly mutations in its zinc finger [17]) could still effectively inhibit HCC cell proliferation, the inhibitory effect of Nur77 2G was significantly impaired as compared with that of wild-type Nur77 (Supplementary Fig. 1a), implying that Nur77 may also directly regulate the transcription of its downstream target genes to suppress HCC cell proliferation. LncRNAs are involved in the tumorigenesis and metastasis of HCC [6], but related reports about whether Nur77 regulates lncRNAs are rare. To determine whether Nur77 is involved in the regulation of lncRNAs expression, we conducted a lncRNA microarray analysis in control and Nur77-overexpressing Huh7 HCC cells and found that the expression levels of many lncRNAs were changed with Nur77 overexpression. Among those Nur77-regulated lncRNAs, WFDC21P is one of the most greatly upregulated lncRNA (Fig. 1a), and this upregulation of WFDC21P by Nur77 could be consistently verified in Huh7, HepG2, and PLC HCC cell lines (Fig. 1b). When Nur77 were knocked down, the WFDC21P expression level significantly decreased in these three HCC cell lines (Fig. 1c). Moreover, the expression of WFDC21P was positively correlated with that of Nur77 in L02 human hepatocyte and eight HCC cell lines (Fig. 1d), but not in ten non-liver cancer cell lines (Supplementary Fig. 1b). Therefore, these results indicate the specifically positive regulation of lncRNA–WFDC21P by Nur77 in HCC.

WFDC21P (NCBI Reference Sequence: NR_030732.1) is located on chromosome 17 in humans and composed of three exons. No WFDC21P homolog was found in mouse or rat. Both the coding potential calculator (CPC) score and GeneID score suggested that WFDC21P has no protein coding potential (Supplementary Fig. 1c) [22, 23], which is consistent with the results of the evaluation by PhyloCSF (Supplementary Fig. 1d) [24]. The full length of WFDC21P (621 nt) was then cloned and ligated into mammalian expression vectors in frame with the flag tag. Although transfection of these WFDC21P constructs into Huh7 or HepG2 cells led to high RNA expression levels of WFDC21P, no flag-tag protein was detected (Supplementary Fig. 1e), further supporting the results of CPC, Gene ID, and PhyloCSF.

In Huh7 or HepG2 cells, Nur77 is located in both the nuclear and cytosolic fractions (Supplementary Fig. 1f). Based on the fact that Nur77 is a transcriptional factor [25], we proposed that Nur77 may transcriptionally regulate WFDC21P. By searching for the promoter sequence of WFDC21P, two conserved NBREs (AAAGGTCA/TGACCTTT) that constitute the DNA binding element for Nur77 were identified [19]. We then constructed several WFDC21P promoter mutants, as indicated in Fig. 1e, for the luciferase assay. Overexpression of Nur77 in Huh7 cells effectively increased the activity of the WFDC21P promoter; in contrast, transfection of the Nur77 2G mutant failed to increase WFDC21P promoter activity (Fig. 1f), implying that the ability to bind DNA is required for Nur77 to regulate WFDC21P. Promoter deletion analysis revealed that when both the proximal and distal NBREs in the WFDC21P promoter were deleted, Nur77 could no longer activate the WFDC21P promoter (Fig. 1g). To further verify whether the NBREs are required for Nur77 regulation of WFDC21P promoter activity, the two NBREs were mutated, respectively (Mut1 or Mut2) or in combination (Mut3). As expected, Nur77 activated the wild-type WFDC21P promoter, moderately enhanced the activity of the Mut1 and Mut2 promoters, but was unable to regulate the activity of the Mut3 WFDC21P promoter (Fig. 1h, left). Consistent with this finding, mutation of the proximal NBRE on WFDC21P promoter 1 (Mut4) abolished the effect of Nur77 on its activity (Fig. 1h, right). The localization of Nur77 to the WFDC21P promoter was then further confirmed by a chromatin immunoprecipitation assay, which indicated that Nur77 could bind to both the proximal and distal NBREs in Huh7 and HepG2 cells (Fig. 1i). Therefore, these data suggested that Nur77 binds to the NBREs on WFDC21P promoter to transcriptionally activate lncRNA–WFDC21P expression in HCC cells.

### WFDC21P suppresses HCC cell proliferation and tumor growth

**Fig. 2:**
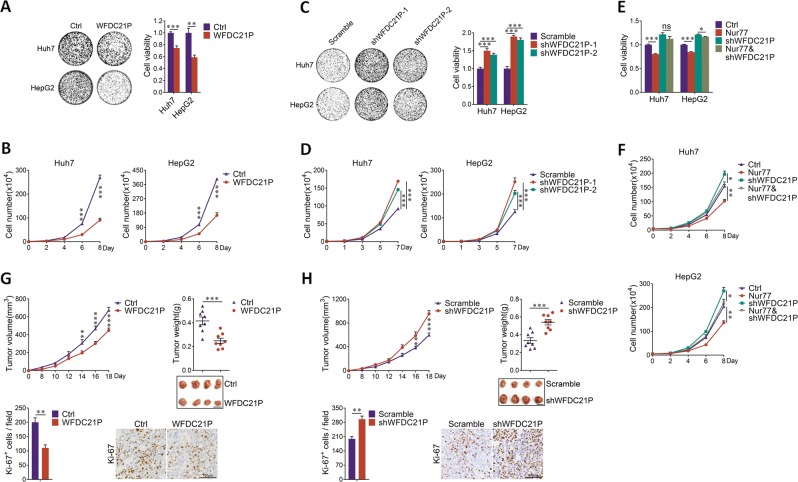
WFDC21P impedes HCC cell proliferation and HCC development. **a** and **b** Overexpression of WFDC21P suppresses HCC cell proliferation. WFDC21P was overexpressed in Huh7 and HepG2 cells. Colony formation assays (**a**, left), MTT assays (**a**, right) were performed, and cell growth curves (**b**) were generated by cell counting analysis. **c**, **d** Knocking down WFDC21P promotes HCC cell proliferation in Huh7 and HepG2 cells. Two different shRNA sequences (shWFDC21P-1 and shWFDC21P-2) were used for WFDC21P knockdown. **e**, **f** WFDC21P is partially required for Nur77 to inhibit HCC cell proliferation. WFDC21P was knocked down by shWFDC21P-1 in Huh7 or HepG2 cells. Nur77 was then overexpressed in control or WFDC21P-knockdown cells. **g** WFDC21P suppresses xenograft tumor growth. Control Huh7 cells or Huh7 cells overexpressing WFDC21P were injected subcutaneously into nude mice to form xenograft tumors (*n* = 8). Tumor growth curve (upper left), tumor weights, and representative xenograft tumor images are shown (upper right). Scale bar, 1 cm. Ki-67 expression was evaluated in the tumor sections by immunohistochemistry and Ki-67 positive cells were quantified in 20 randomly selected fields per mouse (lower). Scale bar, 100 μm. **h** Knocking down WFDC21P enhances xenograft tumor growth. WFDC21P knockdown Huh7 cells were injected subcutaneously into nude mice to form xenograft tumors (*n* = 8). The data are represented as the means ± SEM of at least three independent experiments. *p < 0.05; **p < 0.01; ***p < 0.001 and ns no significance.

We then tested the biological functions of WFDC21P in HCC cells. The ectopic expression of WFDC21P not only suppressed colony formation (Fig. 2a, Supplementary Fig. 2a), but also inhibited cell proliferation in Huh7 and HepG2 cells, as revealed by MTT and cell counting assays (Fig. 2a, b). To further verify this inhibitory effect of WFDC21P, WFDC21P was knocked down by two independent short hairpin RNAs in either Huh7 or HepG2 cells (Supplementary Fig. 2b). As expected, both colony formation and cell proliferation were substantially enhanced upon WFDC21P knockdown (Fig. 2c, d). Importantly, this WFDC21P function was not due to its regulation on cell survival, as WFDC21P has no effect on cell viability or apoptosis in both Huh7 and HepG2 cells (Supplementary Fig. 2c). Hence, WFDC21P suppresses HCC cell proliferation.

Since Nur77 also inhibits HCC growth [21], we hypothesized that WFDC21P may be partially required for Nur77 to exert its inhibitory effect. To address this point, Nur77 was stably expressed in control or WFDC21P-knockdown HCC cells (Supplementary Fig. 2d). We found that Nur77 could efficiently suppress HCC cell proliferation in the control cells, but this inhibitory effect was largely impaired in the WFDC21P-knockdown cells (Fig. 2e, f). Therefore, WFDC21P functions as a downstream target of Nur77 that inhibits HCC cell proliferation. Since Nur77 also regulates PEPCK1 to suppress HCC, we then verified whether there will be any interplay between WFDC21P and PEPCK1. RNA immunoprecipitation (RIP) and the MS2-tagged RNA affinity assay (Supplementary Fig. 2e) revealed that WFDC21P could not interact with PEPCK1 (Supplementary Fig. 2f). Moreover, WFDC21P and PEPCK1 did not influence the expression of each other mutually (Supplementary Fig. 2g, h). Therefore, it is likely that both WFDC21P and PEPCK1 are regulated by Nur77, and they work in isolation to affect HCC growth.

We then tested whether WFDC21P influences tumor growth in vivo. Control Huh7 cells or Huh7 cells stably overexpressing WFDC21P were subcutaneously injected into nude mice to form xenografts. As shown in the tumor growth curves, the overexpression of WFDC21P substantially retarded xenograft tumor growth, which was associated with lower tumor weight and tumor volume (Fig. 2g). Staining of Ki-67, a marker for cell proliferation, in xenograft sections further verified the inhibition of tumor cell proliferation but not induction of apoptosis by WFDC21P (Fig. 2g, Supplementary Fig. 2i). In contrast, knockdown of WFDC21P in Huh7 cells not only markedly accelerated xenograft tumor growth and increased tumor weight (Fig. 2h) but also effectively promoted tumor cell proliferation, as revealed by Ki-67 staining (Fig. 2h). Notably, the overexpression or knockdown efficiency in xenografts could be clearly observed after harvest (Supplementary Fig. 2j). Therefore, these results demonstrate a novel function of WFDC21P in the repression of HCC growth.

### WFDC21P inhibits HCC cell metastasis

**Fig. 3:**
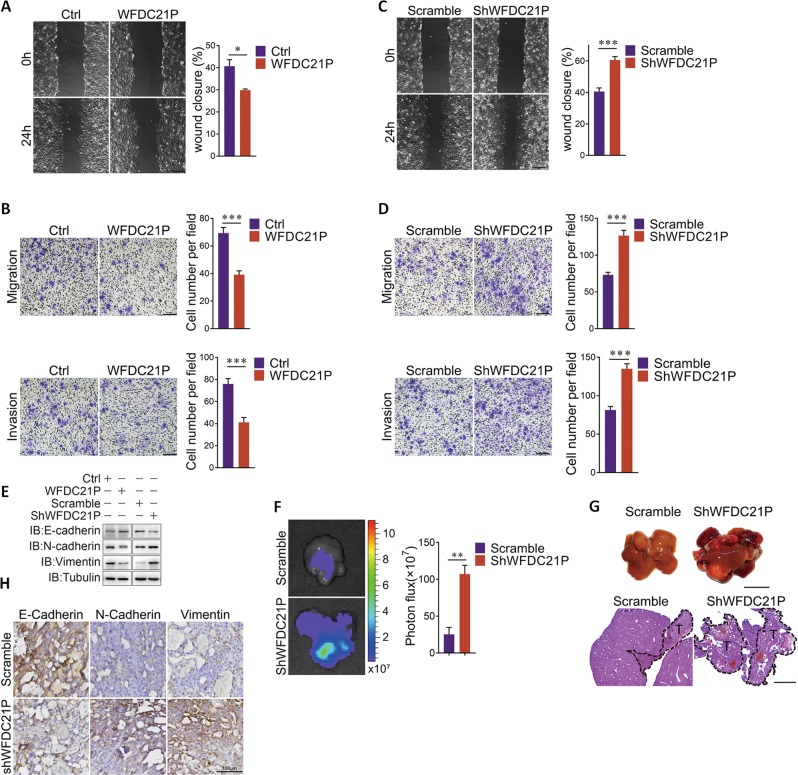
WFDC21P inhibits HCC metastasis. **a**, **b** Overexpression of WFDC21P mitigates the migratory and invasive capacities of Huh7 cells. Wound healing assays (**a**), transwell cell migration assays (**b**, upper), and transwell cell invasion assays (**b**, lower) of control Huh7 cells and WFDC21P overexpressed Huh7 cells were performed with the pretreatment of mitomycin C (10 μg/mL) for 2 h. The gap sizes of wound healing assay were shown, and the cell migration and invasion numbers of at least five fields were counted. Scale bars in **a**, **b** were 500 μm and 200 μm, respectively. **c**, **d** knocking down WFDC21P enhances the migratory and invasive capacities of Huh7 cells. **e** WFDC21P inhibits EMT in HCC cells. WFDC21P was knockdown or overexpressed in Huh7 cells. The protein levels of E-cadherin, N-cadherin, and Vimentin were detected by western blotting. **f**, **g** Depletion of WFDC21P promotes tumor metastasis in mice. Luciferase-expressing control and WFDC21P knocking down Huh7 cells were intrasplenically injected into nude mice (*n* = 6). Liver metastasis was quantified at 58 days after the implantation. Representative bioluminescent images of the mice livers and bioluminescent signal intensities were shown (**f**). Representative images of livers and liver H&E-staining are shown (**g**). Scale bars in **f**, **g** were 1 cm and 2 mm, respectively. **h** WFDC21P suppresses EMT in tumor tissues. The expressions of E-cadherin, N-cadherin, and Vimentin were detected in metastatic tumor tissues from **g**. Scale bars, 100 μm. The data are represented as the means ± SEM of at least three independent experiments. *p < 0.05; and ***p < 0.001.

To further gain insight into the effect of WFDC21P on HCC metastasis, wound healing assays were carried out in Huh7 cells that were pretreated with mitomycin C to inhibit cell proliferation. Under this circumstance, overexpression of WFDC21P clearly weakens the ability of Huh7 cells for migration (Fig. 3a, Supplementary Fig. 3a). Similar results were also achieved by transwell assays, in which the migration and invasion of Huh7 cells were obviously inhibited upon the overexpression of WFDC21P (Fig. 3b). In contrast, the wound healing, invasive, and migratory capacities of Huh7 cells were enhanced when WFDC21P was knocked down in Huh7 cells (Fig. 3c, d, Supplementary Fig. 3b). Hence, WFDC21P is able to inhibit HCC cell metastasis. This WFDC21P effect was closely related with the inhibition of epithelial–mesenchymal transition, as knockdown of WFDC21P clearly inhibited the expression of E-cadherin, while enhancing N-cadherin and vimentin expressions (Fig. 3e). In contrast, overexpression of WFDC21P enhanced E-cadherin level and suppressed N-cadherin and vimentin expressions (Fig. 3e).

The effect of WFDC21P on HCC cell metastasis was further evaluated in mouse model. Control and WFDC21P knockdown Huh7 cells with luciferase expression were intrasplenically injected into nude mice. Compared with control Huh7 cells, knocking down WFDC21P accelerated the hepatic metastases as accessed by monitoring the bioluminescent signal intensities (Fig. 3f), which is closely associated with the increase in the liver metastases burden (Fig. 3g). Consistently, E-cadherin expression in tumor tissues was suppressed by knocking down WFDC21P, while the expressions of N-cadherin and vimentin were enhanced (Fig. 3h). Taken together, these results indicated the inhibitory role of WFDC21P in HCC metastasis both in vitro and in vivo.

### WFDC21P is lowly expressed in human HCC tissues

**Fig. 4:**
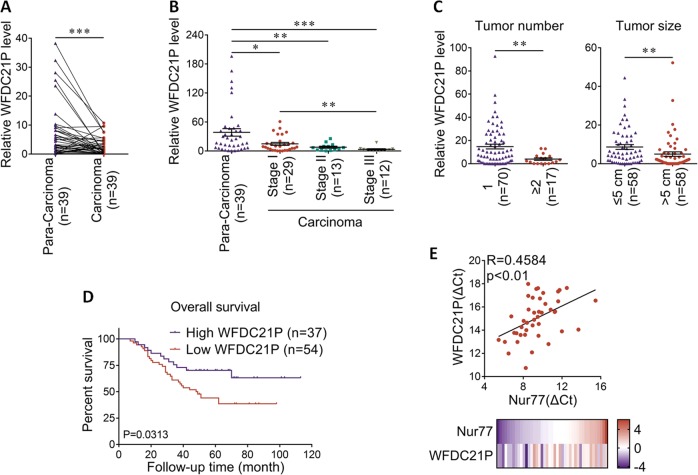
WFDC21P inhibits HCC development in clinical samples. **a** WFDC21P is lower expressed in carcinoma tissues than in paired paracarcinoma tissues (*n* = 39). WFDC21P level was detected by RT-qPCR. **b** WFDC21P expression levels are decreased as HCC progress. WFDC21P expression levels in 39 paracarcinoma samples and 54 HCC samples grouped into stages I–III were detected. **c** WFDC21P expression levels in patients with tumor numbers of 1 or ≥2 (*n* = 87) and tumor sizes of ≤5 or >5 cm (*n* = 116) were detected by RT-qPCR. **d** The positive correlation between overall survival of HCC patients and WFDC21P expression levels. Survival information of 91 patients is available. **e** Correlation chart and heat map show the positive correlation between Nur77 and WFDC21P in clinical HCC and paracarcinoma tissues (*n* = 43). The expression levels of Nur77 and WFDC21P were examined by RT-qPCR. The data are represented as the means ± SEM of at least three independent experiments. *p < 0.05; **p < 0.01 and ***p < 0.001.

To further extend the role of WFDC21P to the pathological level, the expression level of WFDC21P in clinical HCC tissue samples was determined by RT-qPCR. WFDC21P was more highly expressed in paracarcinoma samples than in its paired carcinoma samples (Fig. 4a). With the progression of HCC from stage I to III, WFDC21P expression gradually decreased (Fig. 4b, Supplementary Fig. 4a). Importantly, the expression level of WFDC21P in clinical samples was not only inversely correlated with the number of tumor nodules and tumor size (Fig. 4c, Supplementary Tables 1 and 2), but also closely associated with the overall survival in HCC patients (Fig. 4d). Therefore, these data imply the inhibitory roles of WFDC21P in the pathogenesis and prognosis of HCC.

We found that the Nur77 mRNA level was decreased in carcinoma samples than in its paired paracarcinoma samples, and this Nur77 expression further decreased with the progression of HCC (Supplementary Fig. 4b–d), in line with our previous report that Nur77 protein level was decreased in clinical HCC samples [21]. Notably, the expression of WFDC21P was positively correlated with that of Nur77 in HCC samples (Fig. 4e). Therefore, these results from clinical tumor samples support the notion that WFDC21P is a downstream target of Nur77, and functions as a tumor suppressor in HCC.

### WFDC21P participates in the regulation of glycolysis

**Fig. 5:**
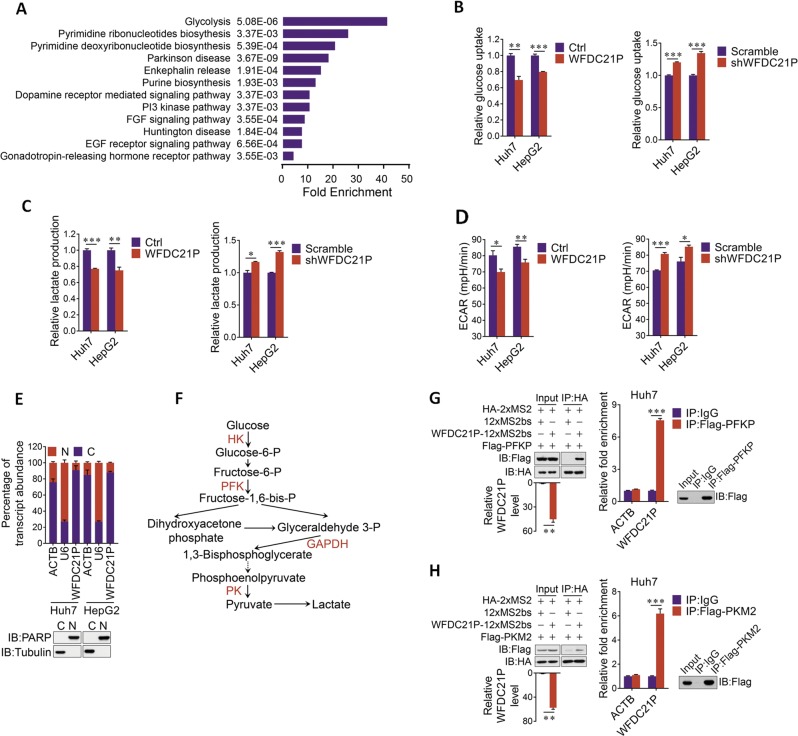
WFDC21P suppresses glycolysis in HCC cells. **a** Gene ontology enrichment analysis for proteins pull down by WFDC21P and identified by mass spectrometry. The significantly enriched pathways with their FDR adjusted *p* values were shown. **b** WFDC21P attenuates glucose uptake. The glucose uptake levels of Huh7 and HepG2 cells with WFDC21P overexpression (left) or WFDC21P knockdown (right) were determined. **c** WFDC21P decreases lactate production. The lactate production levels of Huh7 and HepG2 cells with WFDC21P overexpression (left) or WFDC21P knockdown (right) were determined. **d** WFDC21P suppresses the glycolytic capacity. The extracellular acidification rates (ECARs) of Huh7 and HepG2 cells with WFDC21P overexpression (left) or WFDC21P knockdown (right) were shown. The ECAR value represents the glycolytic capacity. **e** WFDC21P is mainly located in the cytoplasm. WFDC21P levels were detected in the nuclear (N) or cytoplasmic (C) fractions of Huh7 and HepG2 cells. U6 and β-actin (ACTB) were used as nuclear and cytoplasmic RNA markers. PARP and tubulin were considered nuclear (N) and cytoplasmic (C) marker proteins, respectively. **f** A schematic diagram of the glycolytic pathway. **g**, **h** WFDC21P interacts with PFKP (**g**) and PKM2 (**h**) as determined by protein immunoprecipitation using the MS2/MS2bs system (left) or RIP experiments (right). The expression of WFDC21P and the efficiency of immunoprecipitation were determined by RT-qPCR and western blotting, respectively. The data are represented as the means ± SEM of at least three independent experiments. *p < 0.05; **p < 0.01 and ***p < 0.001.

It is well known that lncRNAs function via interacting with various proteins [8]. To clarify the underlying mechanism for the inhibitory effect of WFDC21P in HCC, WFDC21P interacting proteins were pulled down and subjected for mass spectrometry analysis (Supplementary Table 3). Gene ontology enrichment analysis with these WFDC21P interacting proteins revealed that the glycolysis pathway was dramatically enriched (Fig. 5a), indicating that WFDC21P may regulate glycolysis through binding to the proteins involved in glycolysis. Since tumor cells preferentially use glycolysis for energy supplementation to sustain their proliferation and aggressive behavior [26], and Nur77 effectively inhibited glycolysis in HCC cells (Supplementary Fig. 5a, b), we hypothesized that WFDC21P, as a downstream target of Nur77, may regulate glycolysis to influence HCC cell proliferation. Expectedly, the ectopic expression of WFDC21P efficiently inhibited glucose uptake and lactate production in both Huh7 and HepG2 cells (Fig. 5b, c, Supplementary Fig. 5c). Reciprocally, WFDC21P knockdown in those cells resulted in increased glucose uptake and lactate production compared with that of the controls (Fig. 5b, c, Supplementary Fig. 5d). The extracellular acidification rate (ECAR) in Huh7 and HepG2 was then measured and the results indicated that cells with ectopic WFDC21P expression had a decreased glycolytic capacity. In contrast, knockdown of WFDC21P increased the glycolytic capacity compared with that of the controls (Fig. 5d). These data thus suggest that WFDC21P may inhibit HCC development by modulating glycolysis.

A subcellular distribution analysis revealed that WFDC21P was mainly localized in the cytoplasm (Fig. 5e). We then further verified the interaction of WFDC21P with the key enzymes in glycolysis (Fig. 5f) [27], which also occurs in cytoplasm. RIP and the MS2-tagged RNA affinity assay revealed that WFDC21P could interact strongly with PFKP (Fig. 5g), the main phosphofructokinase (PFK) isoform in HCC cells (Supplementary Fig. 5e), and PKM2 (Fig. 5h), while it demonstrated faint interaction with GAPDH and no interaction with hexokinase 2 (HK2) (Supplementary Fig. 5f, g), in line with the results obtained by mass spectrometry. Moreover, a series of deletion mapping analyses revealed that the exon 3 of WFDC21P (from 421–621 nt) was responsible for the interaction with PFKP, while the exon 2 (from 272–420 nt) was important for PKM2 interaction (Supplementary Fig. 5h, i). Therefore, these results suggest that WFDC21P interacts with two rate-limiting enzymes in glycolysis, thereby inhibiting glycolysis.

### WFDC21P regulates the activity of PFKP and PKM2

**Fig. 6:**
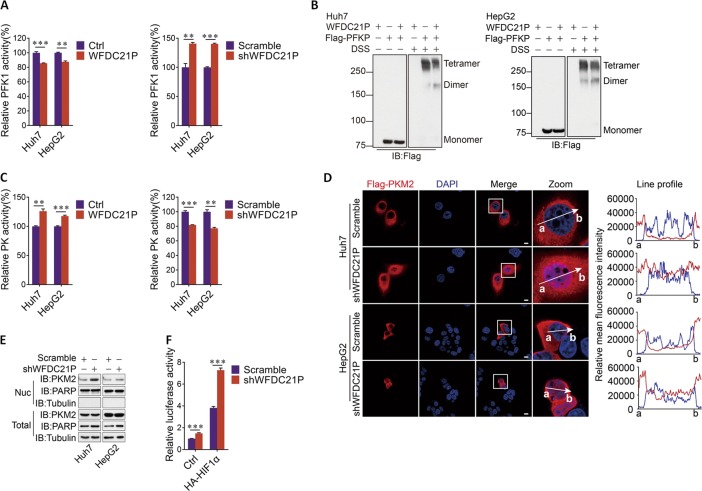
WFDC21P regulates the activities of PFKP and PKM2. **a** WFDC21P inhibits PFKP activity. PFK1 activity was measured in WFDC21P overexpressing (left) or knocking down (right) Huh7 and HepG2 cells. **b** WFDC21P inhibits the tetramer formation of PFKP. Huh7 or HepG2 cells were transfected with or without WFDC21P and PFKP, the PFKP polymers were then detected. **c** WFDC21P enhances PKM2 activity. The PK activity was measured in WFDC21P overexpressing (left) or knocking down (right) Huh7 and HepG2 cells. **d**, **e** Knockdown of WFDC21P promotes PKM2 nuclear translocation. The localization of PKM2 was detected by immunofluorescent staining (**d**) or fractionation assays (**e**) in control or WFDC21P-knockdown Huh7 and HepG2 cells. The line profiles of Flag-PKM2 and DAPI signal intensities are shown. Scale bar, 10 μm. **f** Knocking down WFDC21P enhances the transcriptional activity of HIF1α. The transcriptional activity of HIF1α was determined using a hypoxia response element (HRE) reporter in luciferase assays in control or WFDC21P-knockdown Huh7 cells. The data are represented as the means ± SEM of at least three independent experiments. **p < 0.01 and ***p < 0.001.

We then determined whether WFDC21P alters the activities of these metabolic enzymes. WFDC21P had almost no effect on the enzyme activity of GAPDH, although it could slightly interact with GAPDH (Supplementary Figs. 5g, 6a–d). However, stably expressing WFDC21P in both Huh7 and HepG2 cells decreased PFK1 activity compared with the control (Fig. 6a, left). In contrast, PFK1 activity was increased by the knockdown of WFDC21P in these cells (Fig. 6a, right). The interaction between WFDC21P and PFKP was required for this inhibitory effect of WFDC21P, as deletion of exon 3 in WFDC21P abolished the regulation of WFDC21P on PFK1 activity (Supplementary Fig. 6e). As a result, the inhibitory effects of WFDC21P-ΔE3 on glycolysis and cell proliferation were impaired (Supplementary Fig. 6f, g). Because tetramer formation is critical for the enzyme activity of PFK1 [28], we then tested whether WFDC21P influences PFKP polymerization. As expected, although the overexpression of WFDC21P did not influence the expression level of PFKP, it clearly impaired the tetramer formation of PFKP in both Huh7 and HepG2 cells (Fig. 6b, Supplementary Fig. 6h). Evidently, WFDC21P interacts with PFKP to impede the tetramer formation of PFKP, leading to the suppression of PFK1 activity.

We then tested the influence of WFDC21P on PKM2 activity. PKM2 activity was efficiently enhanced upon WFDC21P overexpression but suppressed by WFDC21P knockdown (Fig. 6c). This regulation was dependent on the WFDC21P–PKM2 interaction. When the interaction of WFDC21P with PKM2 was abolished, the effects of WFDC21P-ΔE2 on PKM2 activity, glycolysis, and cell proliferation were also impaired (Supplementary Fig. 6i–k). Since the enhancement of PKM2 activity is beneficial for tumor suppression [26], the WFDC21P–enhanced PKM2 activity may thus support the tumor inhibitory function of WFDC21P. PKM2 mainly exerts its enzyme activity in the cytoplasm [29]. However, knockdown of WFDC21P markedly induced the translocation of PKM2 from the cytoplasm to the nucleus in both Huh7 and HepG2 cells (Fig. 6d, e), suggesting the retention of PKM2 in the cytoplasm by cytoplasmic WFDC21P. Once translocated to the nucleus, PKM2 could function as a transcriptional coactivator to promote the activity of several oncogenic transcriptional factors, such as HIF1α [30]. Indeed, the nuclear localization of PKM2 induced by WFDC21P knockdown was closely associated with the enhancement of HIF1α transcriptional activity in Huh7 cells (Fig. 6f). Therefore, the above results suggested that WFDC21P could reverse the Warburg effect in HCC by modulating the activity of PFKP and PKM2.

### WFDC21P inhibits tumor growth by regulating PFKP and PKM2

**Fig. 7:**
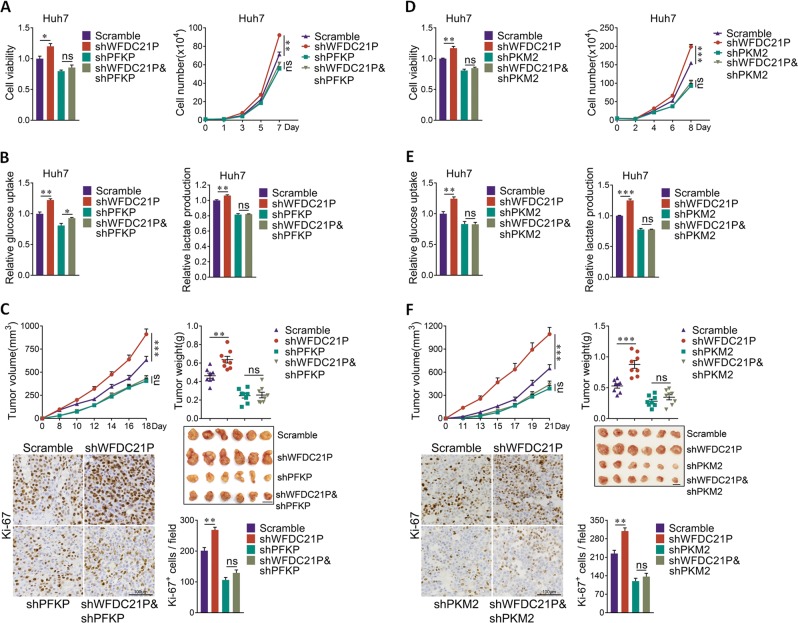
WFDC21P inhibits cell proliferation and tumorigenesis via PFKP and PKM2. **a** WFDC21P cannot influence cell proliferation in PFKP-knockdown cells. WFDC21P was knocked down in control Huh7 cells or Huh7 cells with silenced PFKP. MTT assays (left) and cell counting analysis (right) were carried out. **b** WFDC21P cannot inhibit glucose uptake and lactate production in PFKP-knockdown cells. Glucose uptake (left) and lactate production (right) were detected in cells descripted in **a**. **c** WFDC21P cannot inhibit tumor growth in xenografts derived from cells with PFKP knocked down. The tumor growth curves, tumor weights and Ki-67 expression levels are shown. Scale bars in xenograft images and Ki-67 staining were 1 cm and 100 μm, respectively. **d** WFDC21P cannot influence cell proliferation in PKM2-knockdown cells. **e** WFDC21P cannot inhibit glucose uptake and lactate production in PKM2-knockdown cells. **f** WFDC21P cannot inhibit tumor growth in xenografts derived from cells with PKM2 knocked down. The tumor growth curves, tumor weights and Ki-67 expression levels are shown. Scale bars in xenograft images and Ki-67 staining were 1 cm and 100 μm, respectively. The data are represented as the means ± SEM of at least three independent experiments. *p < 0.05; **p < 0.01; ***p < 0.001 and ns no significance.

The glycolytic regulation was required for Nur77 to suppress HCC cell proliferation, as treatment of 2-DG, an inhibitor for glycolysis, almost abolished Nur77 effect on cell proliferation (Supplementary Fig. 7a, b). We then explored whether WFDC21P, as a downstream target of Nur77, suppresses HCC tumorigenesis by the regulation of two glycolytic enzymes PFKP and PKM2. It was demonstrated that the knockdown of WFDC21P could efficiently enhance cell proliferation in Huh7 cells. However, when PFKP was knocked down simultaneously, WFDC21P lost its ability to regulate cell proliferation (Fig. 7a, Supplementary Fig. 7c). Consistent with this finding, the knockdown of WFDC21P in PFKP-knockdown Huh7 cells could hardly promote glucose uptake and lactate excretion (Fig. 7b). We further investigated whether WFDC21P regulates HCC tumorigenesis by targeting PFKP in xenograft tumor models. In accordance with the results at the cellular level, knockdown of WFDC21P substantially promoted tumor growth and tumor cell proliferation in tumors derived from control cells but not in those derived from Huh7 cells with PFKP knocked down (Fig. 7c). Hence, WFDC21P inhibits HCC by regulating PFKP.

The involvement of PKM2 in WFDC21P-inhibited HCC was also tested. As expected, WFDC21P could suppress cell proliferation, glucose uptake and lactate production in control cells but not in PKM2-knockdown cells (Fig. 7d, e, Supplementary Fig. 7d). The in vivo xenograft tumor experiments also demonstrated that the knockdown of PKM2 abolished the inhibitory effects of WFDC21P on tumor growth (Fig. 7f), further demonstrating the importance of the WFDC21P–PFKP/PKM2 axis in HCC inhibition. In summary, these results demonstrate a novel crosslink between WFDC21P and glycolysis, in which WFDC21P interacts with PFKP and PKM2 to regulate their activities, leading to the obstruction of the Warburg effect and the inhibition of HCC.

### Nur77 agonist Csn-B induces WFDC21P expression to suppress HCC

**Fig. 8:**
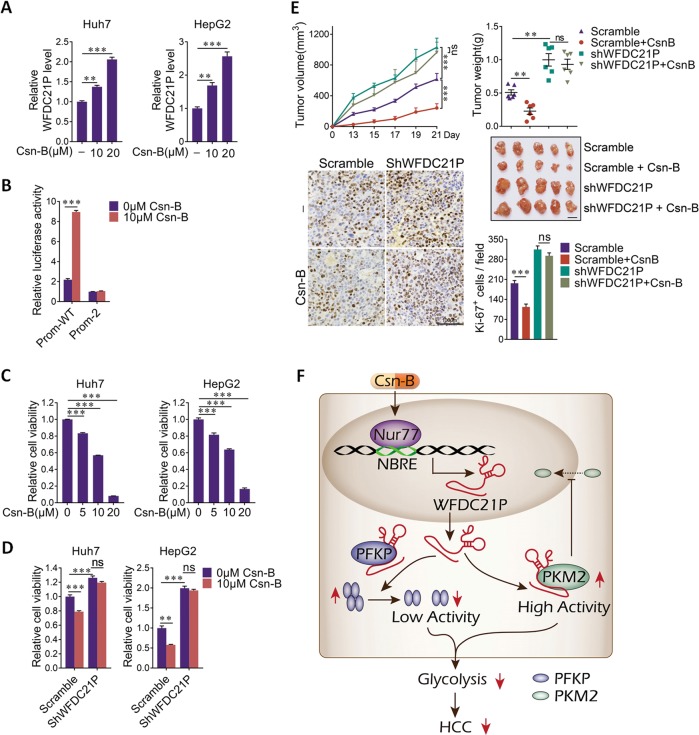
WFDC21P is required for Csn-B-inhibited HCC cell proliferation. **a** Csn-B increases WFDC21P expression levels in a dose dependent manner in Huh7 and HepG2 cells. The WFDC21P expression levels were detected by RT-qPCR. **b** Csn-B increases the activity of the WFDC21P promoter. Luciferase assays were performed in Huh7 cells. **c** Csn-B inhibits the HCC cell viability. MTT assays were performed in Huh7 and HepG2 cells. **d** Csn-B cannot inhibit cell viability in WFDC21P knockdown cells. MTT assays were performed in Huh7 and HepG2 cells. **e** WFDC21P is required for the inhibitory effect of Csn-B in xenograft tumors (*n* = 6). The tumor growth curves, tumor weights, and Ki-67 expression levels are shown. Scale bars in xenograft images and Ki-67 staining were 1 cm and 100 μm, respectively. **f** Schematic diagram of the Nur77–WFDC21P–PFKP/PKM2 axis in HCC progression inhibition. Csn-B, an agonist for Nur77, stimulates the transcriptional activity of Nur77. Nur77 then activates the transcription of WFDC21P by directly binding to NBREs on the promoter of WFDC21P. On the one hand, WFDC21P interacts with PFKP to attenuate its tetramer formation, resulting in decreased PFKP activity. On the other hand, WFDC21P interacts with PKM2, which blocks its nuclear translocation and, therefore, its function as a transcriptional coactivator. As a result, WFDC21P inhibits glycolysis in HCC cells and suppresses HCC development. *p < 0.05; **p < 0.01; ***p < 0.001 and ns no significance.

Cytosporone B (Csn-B) is an agonist of Nur77, which specifically stimulates the transcriptional activity of Nur77 by binding to the ligand-binging domain of Nur77 [31]. We then tested whether Csn-B could stimulate WFDC21P expression to suppress HCC. As expected, Csn-B substantially elevates the expression level of WFDC21P in Huh7 and HepG2 cells in a dose dependent manner (Fig. 8a), and this effect of Csn-B is functioned through promoting the promoter activity of WFDC21P (Fig. 8b). When the Nur77 response elements in WFDC21P promoter were deleted, Csn-B can no longer stimulate WFDC21P promoter activity (Fig. 8b). As a result, Csn-B treatment effectively suppressed the growth of Huh7 and HepG2 cells in a WFDC21P dependent manner (Fig. 8c, d).

This WFDC21P dependent effect of Csn-B was further verified in xenograft mouse model. It was demonstrated that Csn-B treatment evidently suppressed the tumors growth and tumor cell proliferation in control Huh7 cells formed xenograft, but not in xenograft tumors derived from WFDC21P knockdown Huh7 cells (Fig. 8e). Hence, Csn-B, which upregulates WFDC21P to inhibit HCC, may represent an attractive therapeutic drug for HCC treatment.

## Discussion

Although some nongenomic actions of Nur77 in HCC development and progression have been reported in recent years [21, 32], the regulatory roles of Nur77 as a transcriptional factor in HCC have remained elusive. Here, we found that Nur77 transcriptionally activates the expression of lncRNA-WFDC21P in HCC cells through specifically binding to the promoter region of WFDC21P. WFDC21P markedly inhibited HCC cell proliferation and metastasis via modulating the process of glycolysis, leading to suppressed glucose uptake and lactate production. WFDC21P specifically binds to PFKP and PKM2, key enzymes that catalyze two irreversible committed steps of glycolysis. These interactions not only impede the enzyme activity of PFKP through disrupting its tetramer formation but also suppress the function of PKM2 as a transcriptional coactivator through prohibiting its nuclear translocation. In clinical HCC samples, the expression of WFDC21P is suppressed, and this WFDC21P expression not only positively correlates with that of Nur77, but also closely associated with good prognosis of HCC patients. Moreover, Csn-B, an agonist of Nur77, efficiently inhibits liver cancer growth through inducing the expression of WFDC21P (Fig. 8f). Therefore, the collective evidence from the experiments in our study reveals a novel Nur77 downstream target gene, lncRNA-WFDC21P, and connects the glycolytic remodeling of HCC with the Nur77–WFDC21P–PFKP/PKM2 signaling axis.

Glycolytic remodeling is one of the hallmarks of various tumors, including HCC, and it correlates with tumor progression and worse clinical outcomes [33, 34]. Recently, the involvement of lncRNAs in the regulation of glycolysis has attracted extensive attention. It has been reported that lincRNA-p21 can bind to HIF1α and VHL, thereby disrupting the VHL-HIF1α interaction and causing HIF1α accumulation, which leads to the promotion of glycolysis under hypoxic conditions [35]. In addition, lncRNA prostate cancer gene expression marker 1 (PCGEM1) physically interacts with c-Myc, promotes the chromatin recruitment and the transactivational activity of c-Myc, resulting in c-Myc-dependent metabolic reprogramming [36]. These reports demonstrate the indirect regulation of glycolysis by lncRNAs at the transcriptional level. Whether lncRNAs control glycolysis directly through interacting with glycolytic enzymes is still poorly understood. Although many enzymes in glycolysis catalyze reversible reactions, three regulatory enzymes, namely, hexokinase (HK), PFK, and pyruvate kinase (PK), control the irreversible steps and are considered rate-limiting enzymes [26]. Here, we report that glycolysis pathway was substantially enriched in the WFDC21P-interacting proteins, and at least two key enzymes of glycolysis were directly regulated by WFDC21P, further demonstrating the complexity and flexibility of metabolic regulation by lncRNAs.

WFDC21P inhibits glycolysis by simultaneously interacting with PFKP and PKM2. WFDC21P interacts with PFKP to suppress its catalytic activity. PFK1 is considered the gatekeeper of glycolysis because it catalyzes the step that commits glucose to being catabolized. There are three isoforms of PFK1 in humans in the platelets (PFKP), muscles (PFKM), and liver (PFKL) [37]. Although PFKL is the major type in human liver tissue [38], we demonstrated that PFKP is the primarily expressed PFK1 isoform in HCC cells, which is in line with the results of previous reports and implies that PFKP plays an important role in the metabolic reprogramming of HCC [39]. PFKP exerts its catalytic function in a tetrameric form, which is composed of a dimer of dimers [40]. Because the interface between the two dimers is relatively small [41], it is not surprising that the interaction of WFDC21P with PFKP effectively suppresses tetramer but not dimer formation, leading to the suppression of PFKP activity. On the other hand, WFDC21P also regulates the activity of PKM2. PKM2 is a specific pyruvate kinase isoform that is highly expressed in tumors [42]. Although controversy remains with the functions of different pyruvate kinase isoforms in cancer [43, 44], it is believed that the relatively lower activity level of PKM2 compared with other pyruvate kinase isoforms can support tumor progression by promoting anabolic processes and antioxidant responses [26, 45, 46]. Moreover, PKM2 also functions as a coactivator in the nucleus to promote the transcriptional activities of many oncogenic factors, such as HIF1α, which further facilitates the glycolytic process [47]. The interaction of WFDC21P with PKM2 retains PKM2 in the cytoplasm, which not only inhibits its effect on HIF1α activation but also promotes its catalytic activity, contributing to the suppression of HCC. Therefore, WFDC21P inhibits glycolysis in HCC through regulating PFKP and PKM2 simultaneously.

Our previous finding indicated that Nur77 could activate gluconeogenesis in HCC cells by interacting with and stabilizing PEPCK1 in the cytoplasm, thereby counteracting the glycolysis process and inhibiting HCC growth [21]. However, the detailed mechanism underlying the inhibition of glycolysis by Nur77 remains to be replenished. In this study, we found that the transcriptional activation of WFDC21P by Nur77 also contributes to Nur77-induced inhibition of glycolysis. Nur77 directly binds to two NBREs on the WFDC21P promoter to activate its transcription. Thus, the dual regulatory effect of Nur77 on glycolysis implicates the crucial role played by Nur77 in HCC inhibition. Furthermore, Csn-B, a Nur77 agonist that specifically activate Nur77 transcriptional activity [31], could suppress HCC growth via stimulating WFDC21P expression. Notably, WFDC21P depletion almost totally abolished the inhibitory effect of Csn-B on HCC growth, which indicated that WFDC21P is required for Nur77 to suppress HCC through transcriptional regulation. Since the expression levels of both Nur77 and WFDC21P are suppressed in clinical HCC samples and are negatively correlated with the progression of HCC, we propose that this Nur77–WFDC21P–PFKP/PKM2 axis may contain promising intervention targets for HCC therapy, and Csn-B or other Nur77 agnoists [48–50] may represent effective therapeutic compounds.

## Materials and methods

### Cell culture and clinical tumor cDNA samples

HepG2 cells were obtained from American Type Culture Collection (Manassas, VA, USA). Huh7 and PLC cells were obtained from Cell Bank in the Chinese Academy of Sciences (Shanghai, China). These cell lines were authenticated using short tandem repeat profiling analysis by Guangzhou Cellcook Biological Science and Technology Ltd. Cells were cultured in Dulbecco’s modified Eagle’s medium supplemented with 10% fetal bovine serum (FBS; Gemini, West Sacramento, CA, USA), penicillin (100 U), and streptomycin (100 μg/mL) (all are from BBI, Shanghai, China), maintained at 37 °C in an atmosphere containing 5% CO_2_. Cell lines were kept frozen in liquid nitrogen and after they were thawed, <30 of passages were used for 3 months in the current experiments. All the cell lines were periodically tested for Mycoplasma contamination and were found to be Mycoplasma free.

Liver cancer and adjacent liver tissue cDNA microarrays with patient diagnosis information were obtained from Shanghai Outdo Biotech Company, China.

### Glucose uptake and lactate production assay

For the glucose uptake assay, cells were seeded in 12-well plates and then treated with 10 μM 2-NBDG for 1 h at 37 °C. The uptake of 2-NBDG was analyzed with an FC500 Flow Cytometer (Beckman Coulter, Brea, CA, USA).

For the lactate production assay, cells were seeded in 12-well plates. After culturing in DMEM without FBS for 12 h, the culture media were collected. An aliquot of 540 μL of each sample with 60 μL of D_2_O was added to 5-mm magnetic resonance tubes, and data were collected using a Bruker Avance III 600 MHz NMR magnet system (Bruker, Billerica, MA, USA).

### Metabolic assays

The ECARs of the cells were measured with a Seahorse Biosciences XF96 analyzer (North Billerica, MA, USA). Cells were plated in XF96 plates and then acclimatized at 37 °C for 1 h in XF Base Medium with 2 mM glutamine. Measurements were performed under basal conditions and in response to 10 mM glucose, 5 μM oligomycin, and 100 mM 2-deoxyglucose (all from Sigma-Aldrich). Experiments using the Seahorse system were performed with the following assay conditions: 3 min of mixture, 3 min of waiting, and 3 min of measurement. The ECAR values represent the glycolytic capacity and are obtained by subtracting the nonglycolytic acidification from the maximum value.

### Chromatin immunoprecipitation (ChIP) assay

Cells were crosslinked with 1% formaldehyde for 10 min in culture medium and neutralized using glycine. After wash three times in cold PBS, the Huh7 and HepG2 cell pellets were collected and resuspended in SDS lysis buffer (1% SDS, 10 mM EDTA, and 50 mM Tris-HCl (pH 8.1)) supplemented with protease inhibitors and then sonicated and centrifuged. DNA–protein complexes were immunoprecipitated with the antibody against Nur77 at 4 °C and recovered with protein A/G-Sepharose beads. Rabbit IgG (Sigma-Aldrich) was used as a negative control. Crosslinking was reversed by incubation overnight at 65 °C. DNA was recovered by phenol/chloroform extraction and ethanol precipitation and then subjected to qPCR. The amplification of the GAPDH promoter was used as a negative control.

The specific primers used were as follows:

Distal NBRE: forward, 5′-CTGTTTCCCTTGGCTTAGAG-3′

reverse, 5′-TATGATTTATGTCTTCCCTCCCAG-3′

Proximal NBRE: forward, 5′-TGACATTTAGGCTGGGCCTT -3′

reverse, 5′-GGGAGAGACCCACTCTGTTC -3′

Adjacent region: forward, 5′- CCATAGGTACCTGCGTCGAG-3′

reverse, 5′- CCTGTCCTTACCCTGCAACA - 3′

GAPDH promoter: forward, 5′- TACTAGCGGTTTTACGGGCG -3′

reverse, 5′- TCGAACAGGAGGAGCAGAGAGCGA -3′.

### Cell proliferation and colony formation assays

Cell proliferation was measured by MTT assays and cell counting analysis. For the MTT assay, cells were plated in 96-well plates and cultured for 72 h; then, 10 μL of 10 mg/mL MTT stock was added to each well and incubated for another 4 h. After dissolving the cells in dimethyl sulfoxide (DMSO), the absorbance was read with a POLARstar Omega system (BMG Labtech, VIC, Australia). For the cell growth curves generated by cell counting analysis, cells were seeded in six-well plates, and cell numbers were counted every other day after trypan blue staining.

For the colony formation assay, cells were seeded in 60-mm dishes and cultured for 10 days. The colonies were fixed with 4% paraformaldehyde and stained with 0.1% crystal violet in 20% methanol for 15 min, then washed with PBS and photographed.

### Wound healing assays

The Huh7 cells were seeded in six-well plate. After achieving 90–95% confluence, cells were pretreated with mitomycin C (10 μg/mL) for 2 h prior to the scratch. The scratch wounds were generated in the cell monolayers using a plastic pipette tip, and the cells washed and cultured in DMEM without FBS. The migration of the cells at the edge of the wounds was recorded at 0 and 24 h, and the gap size was processed and calculated with Image J software, version 1.52n (National Institutes of Health).

### Cell migration and invasion assays

Matrigel (Corning, Lowell, MA, USA) was diluted with the precooled DMEM without FBS and plated 100 μL in the transwell chamber (8 μm pore, Corning). The transwell chamber uncoated and coated with Matrigel were used for migration and invasion assays, respectively. 1 × 10^5^ cells were pretreated with 10 μg/mL mitomycin C for 2 h, and then suspended in 100 μL of DMEM without FBS and seeded in the upper chamber, the lower chamber was filled with DMEM containing 20% FBS. After incubation at 37 °C for 36 h, the noninvaded or nonmigrated cells were gently removed with cotton swabs, the invaded or migrated cells were fixed and stained using 0.5% crystal violet. The stained cells were photographed and counted.

### Enzyme activity assay

PFK1 activity was determined as described previously [51]. Briefly, the reaction was performed using cell lysates (10 µg) in 1 mL of reaction buffer containing 50 mM Tris-HCl (pH 7.5), 100 mM KCl, 5 mM MgCl_2_, 5 mM Na_2_HPO_4_, 1 mM NH_4_Cl, 0.1 mM AMP, 1 mM ATP, 0.2 mM NADH, 5 mM fructose-6-phosphate, 5 U of triosephosphate isomerase, 1 U of α-glycerophosphate dehydrogenase (Sigma-Aldrich) and 1 U of aldolase (Aladdin, Shanghai, China). Absorbance was recorded at 340 nm at room temperature every 15 s for 10 min using a POLARstar Omega system (BMG Labtech). One unit of PFK1 activity was defined as the amount of enzyme that catalyzes the conversion of 1 µM fructose-6-phosphate to fructose-1,6-bisphosphate per min, and the data are presented as relative PFK1 activity levels.

The PK activity of the cell lysates (4 µg) was measured with a PK assay kit (BioVision, Mounysin View, CA, USA) according to the manufacturer’s instructions.

### DSS crosslinking

DSS crosslinking assays were performed with modifications, as previously described [52]. Briefly, cells were lysed in lysis buffer (12.5 mM HEPES (pH 7.5), 30 mM NaCl, 90 mM NaSCN, 1% NP-40) and centrifuged. Aliquots of the supernatants were crosslinked with or without 1 mM DSS for 5 min at room temperature, and the reactions were quenched by adding 1 M Tris-HCl (pH 7.4) to a final concentration of 50 mM. The samples were then subjected to SDS-PAGE and analyzed by western blotting.

### Animal models

Male nude mice (BALB/C, 6–7 weeks old) were obtained from the Slac Laboratory Animal Center (Shanghai, China) and maintained under specific pathogen-free conditions at the Experimental Animal Center at Xiamen University (Xiamen, China) according to the institutional guidelines. Mice were maintained in 12 h light/12 h dark cycles with free access to food and water. All animal experiments were approved by the Animal Ethics Committee of Xiamen University (acceptance no. XMULAC20120030). Mice were randomized into different groups with approximately equivalent numbers before tumor cell inoculation or drug treatment. Tumor number or tumor weight was evaluated in a blinded manner between each group.

For xenograft tumor mice model, Huh7 cells with specific genes overexpression or knockdown (2 × 10^6^) were subcutaneously injected into the right flanks of nude mice. Tumor growth was recorded with a caliper every other day once the tumors were measurable. The volume of the xenografts was calculated with the following formula: *V* = 1/2 ((length) × (width)^2^). For Csn-B treatment, Csn-B was dissolved in DMSO and then mixed with Tween-80 at 1:1 ratio, further diluted with PBS. Five days after HCC cells inoculation, Csn-B was injected via tail vein at a dosage of 10 mg/kg every three days. After 3 weeks inoculation, the tumors were harvested, photographed, and weighed. Tumor growth was recorded in a blinded manner.

For the hepatic metastases model, control and WFDC21P knockdown Huh7 cells with luciferase expression (1.5 × 10^6^) were suspended in 30 μL PBS and intrasplenically injected into nude mice under anesthesia. Fifty eight days after tumor implantation, hepatic metastases were monitored using IVIS@Lumina II system (Caliper Life Sciences, Hopkinton, MA) after 3 mg of D-luciferin (15 mg/mL in PBS) was intraperitoneally injected.

### Reproducibility and statistical analysis

For cell experiments study, sample size was determined to be adequate based on the magnitude and consistency of measurable differences between groups, usually the number is three or more. For mouse model study, no statistical methods were used to predetermine sample size, which was determined based on previous experimental observations. Sample size for each experiment is indicated in the figure legend.

All statistical analyses were performed using GraphPad Prism 7 (La Jolla, CA, USA). Data are presented as the means ± SEM of at least three independent experiments unless otherwise specified. Significant differences between means were determined with two-tailed Student’s *t* tests or analysis of variance (ANOVA) followed by Tukey’s post hoc test unless otherwise specified. For the WFDC21P expression in clinical samples, nonparametric Mann–Whitney tests or analysis of variance (ANOVA) followed by nonparametric test was employed for comparing categorical variables. The distributions of selected clinical features were compared using the chi-square test. The correlation between the expression levels of WFDC21P and Nur77 was analyzed by Pearson correlation. Kaplan–Meier survival analysis was used to evaluate the relationship between WFDC21P expression and HCC prognosis. The differences were deemed statistically significant at *P* *<* 0.05, highly significant at *P* *<* 0.01, and extremely significant at *P* *<* 0.001.

## Supplementary information

Supplementary information

Supplementary Table 1

Supplementary Table 2

Supplementary Table 3
