# Supplementary material for: Nur77-activated lncRNA WFDC21P attenuates hepatocarcinogenesis via modulating glycolysis
Source: Oncogene. 2020 Jan 20;39(11):2408–23. doi: 10.1038/s41388-020-1158-y (PMC7067692; doi:10.1038/s41388-020-1158-y)

Supplementary information

Supplementary Materials and Methods

Plasmid constructs and transfection

The full length WFDC21P sequence was PCR-amplified and subcloned into pcDNA3.1, pcDNA3.1-Flag (Thermo Fisher Scientific), pcDNA-12 × MS2bs and pLV vectors. Flag-HK2 was gifted by Dr. Qin-xi Li (Xiamen University, Fujian, China). Flag-PFKP, and Flag-GAPDH were gifted by Dr. Sheng-cai Lin (Xiamen University, Fujian, China). The 12 × MS2bs and Flag-2 × MS2 were gifted by Xiao-fei Zheng (Academy of Military Medical Sciences, Beijing, China). HA-2 × MS2 was generated by cloning 2 × MS2 from Flag-2 × MS2 into the pcDNA-HA vector. All constructs were confirmed by DNA sequencing. Transfection was performed using Engreen Entranster-D4000 (Engreen Biosystem Co. Ltd. Beijing, China) transfection reagents according to the manufacturer's instructions, except for 293T cells, which were transfected using the standard calcium phosphate method.

Antibodies and reagents

The anti-HA (cat. #H-9658), anti-Flag (cat. #F-1804), and anti-β-tubulin (cat. #T4026) antibodies were purchased from Sigma-Aldrich (Darmstadt, Germany). The anti-Nur77 (cat. #D63C5), anti-Ki67 (cat. #9027), anti-PKM2 (cat. #3198), anti-PEPCK1 (cat. #12940), anti-N-Cadherin (cat. #13116), anti-Caspase-3 (cat. #9665) and anti-PARP (cat. #9532) antibodies were obtained from Cell Signaling Technology (Danvers, MA, USA). The anti-E-Cadherin (cat. #sc-8462) antibody was obtained from Santa Cruz Biotechnology (Santa Cruz, CA, USA). The anti-Vimentin (cat. #10366-1-AP) antibody

was obtained from ProteinTech Group (Chicago, IL, USA). The anti-Active Caspase-3 (cat. #559565) antibody was obtained from BD Biosciences (San Jose, CA, USA). The anti-GAPDH (cat. #M20006) antibody was obtained from Abmart (Shanghai, China). The chemical reagent 2-(N-(7-nitrobenz-2-oxa-1,3-diazol-4-yl)amino)-2-deoxyglucose (2-NBDG) (cat. #N13195) and Disuccinimidyl suberate (DSS) (cat. #21655) were purchased from Thermo Fisher Scientific (Bremen, Germany). D_2O and 3-(4,5-dimethylthiazol-2-yl)-2,5-diphenyltetrazolium bromide (MTT) were purchased from Sigma-Aldrich. Cytosporone B was previously described.[1] Mitomycin C (cat. #HY-13316) and staurosporine (STS) (cat. #HY-15141) was purchased from MedChemExpress (Shanghai, China).

RNA immunoprecipitation

For immunoprecipitation using the HA-2 × MS2 and 12 × MS2bs system, cells were lysed with Pierce immunoprecipitation (IP) lysis buffer (25 mM Tris-HCl (pH 7.4), 150 mM NaCl, 1% NP-40, 1 mM EDTA, 5% glycerol) containing protease inhibitors and RNase inhibitor (Thermo Fisher Scientific) on ice. After centrifugation, the supernatant of the cell lysates was incubated with the appropriate antibodies for 3 h and then incubated with protein A/G-Sepharose beads for another 1 h at 4°C. The beads were then washed three times with Pierce IP lysis buffer. The pellets were boiled in sample buffer and subjected to western blot or mass spectrometry analysis.

For crosslinked RNA immunoprecipitation, cells were crosslinked with 1% formaldehyde in culture medium and quenched with glycine. Cells were then washed three times with cold phosphate-buffered saline (PBS) and lysed in Pierce IP lysis buffer

containing protease inhibitors and RNase inhibitor. The cell lysates were incubated on ice for 20 min and then sonicated and centrifuged. The supernatants were incubated with the appropriate antibodies for 4 h and then incubated with protein A/G magnetic beads (Cat. #B23202, Bimake, Shanghai, China) for another 1 h, mouse IgG (Sigma-Aldrich) was used as negative control. The beads were washed five times in high-stringency IP lysis buffer containing 500 mM NaCl and resuspended in IP lysis buffer at 70°C for 45 min to reverse the crosslinking. The immunoprecipitated RNA was extracted with TRIzol reagent (Sigma-Aldrich) and subjected to RT-qPCR.

Generation of the lentiviral system

The lentiviral system was generated as previously describe.[2] For WFDC21P overexpression, the full-length or fragment sequence of WFDC21P was subcloned into the lentiviral vector pLV/puro. For the lentiviral knockdown system, the oligonucleotides (Thermo Fisher Scientific) were annealed and subcloned into the lentiviral vector pLL3.7 or LvEX10-luciferase. Nur77, PEPCK1 overexpression and knockdown plasmids were previously established.[2,3] To produce lentiviruses, subconfluent 293T cells were co-transfected with the lentiviral vectors and packaging plasmids using the calcium phosphate transfection method. Lentiviruses were collected 48 h after transfection, filtered through 0.45-μm filters (Millipore, Darmstadt, Germany) and then concentrated by centrifugation at 75,000 × g for 90 min. The virus-containing pellets were dissolved in DMEM. Huh7, PLC and HepG2 cells were infected with the lentiviruses in the presence of 8 μg/mL polybrene (Sigma-Aldrich) and selected with puromycin or hygromycin. The overexpression or knockdown efficiency of the target genes was determined by western

blotting or RT-qPCR.

The oligonucleotide sequences used for the shRNA-targeted RNA were as follows:

shRNA-WFDC21P-1, 5'-GATGTGACTGTCTGGAGTT-3'

shRNA-WFDC21P-2, 5'-GGAGTTCCTTGACTAGGAA-3'

shRNA-PFKP, 5'-GCTGAAGGAGCAATTGATA-3'

shRNA-PKM2, 5'-GCCATAATCGTCCTCACCA-3'

shRNA-Nur77, 5'-GGGCATGGTGAAGGAAGTT-3'

shRNA-PEPCK1, 5'- GACCATCCAGAAGAACACAAT-3'

shRNA-Scramble, 5'-GCGCGCTTTGTAGGATTCG-3'

RNA isolation and quantitative real-time PCR

Total RNA was extracted from cells using TRIzol reagent, and complementary DNA was generated using the FastQuant RT Kit (TIANGEN, Beijing, China) according to the manufacturer's protocol. Quantitative real-time PCR was performed using the SYBR Green Power Master Mix (Promega, Madison, USA) and the CFX96 real-time PCR detection system (Bio-Rad, Berkeley, CA, USA), and the level of actin was used as a normalization control.

The primer sequences for RT-qPCR were as follows:

Nur77: forward, 5'-CTCTGGAGGTCATCCGCAAG-3'

reverse, 5'-CTGGCTTAGACCTGTACGCC-3'

WFDC21P: forward, 5'-CAGGTAAAAACACCAGGCCC-3'

reverse, 5'-GAACTCCAGGAAGGGATGACG-3'

WFDC21P-E1: forward, 5'- AGCGGGTGGAAGGTATAA-3'

reverse, 5'- ATGAGGATCACAGGAGGAG-3'

WFDC21P-E2: forward, 5'- CCGGAGACCACTGTGTCAG-3'

reverse, 5'- GAGGGCCTGGTGTTTTTACCT-3'

WFDC21P-E3: forward, 5'- AAGATCGTCATCCCTTCCT-3'

reverse, 5'- CCACAGGCTTGCTGTTT -3'

PFKP: forward, 5'-GACCTTCGTTCTGGAGGTGAT-3'

reverse, 5'-CACGGTTCTCCGAGAGTTTG-3'

PFKM: forward, 5'-GGTGCCCGTGTCTTCTTTGT-3'

reverse, 5'-AAGCATCATCGAAACGCTCTC-3'

PFKL: forward, 5'-CCTGTACAACCTGTACTCATC-3'

reverse, 5'-CCCATAGTTCCGGTCAAAG-3'

PKM2: forward, 5'-GGGCCATAATCGTCCTCACC-3'

reverse, 5'-GACGAGCTGTCTGGGGATTC-3'

β-actin: forward, 5'-CAGCCTTCCTTCCTGGGCATG-3'

reverse, 5'-ATTGTGCTGGGTGCCAGGGCAG-3'

U6: forward, 5'-GTGCTCGCTTCGGCAGCACA-3'

reverse, 5'-GTGCTCGCTTCGGCAGCACA-3'

Western blotting

Cells were lysed with egg lysis buffer (ELB) (150 mM NaCl, 100 mM NaF, 50 mM Tris-HCl (pH 7.6), 0.5% NP-40 and 1 mM PMSF) supplemented with protease inhibitors. Equal amounts of protein were loaded and separated by sodium dodecyl sulfate-polyacrylamide gel electrophoresis (SDS-PAGE) and then transferred to a polyvinylidene

difluoride membrane (Millipore). The membrane was incubated with the primary antibodies. Horseradish peroxidase (HRP)-linked anti-mouse immunoglobulin G (IgG) or HRP-linked anti-rabbit IgG antibodies were used as secondary antibodies. The immunoreactive products were detected using enhanced chemiluminescence (Pierce, Socochim SA, Lausanne, Switzerland).

Luciferase reporter assay

Cells were transfected with WFDC21P promoter reporter constructs or hypoxia response element (HRE) reporter constructs together with the β-galactosidase (β-gal) expression plasmid. Cells were lysed 24 h after transfection, and luciferase activity was measured and normalized to β-gal activity to determine the transfection efficiency.

Cell survival analysis

WFDC21P was overexpressed or knocked down in Huh7 and HepG2 cells. Twenty four hours later, cells were collected and stained using Annexin V-FITC/PI apoptosis detection kit (eBioscience) according to the manufacturer's instructions. Apoptotic inducer staurosporine (STS) (1 μM) was used as a positive control. The stained cells were analyzed using FC500 flow cytometer (Beckman).

Microarray analysis

Total RNA was amplified and labeled using the Low Input Quick Amp Labeling Kit (Agilent Technologies, Santa Clara, CA, USA) according to the manufacturer's instructions. Labeled cRNA was purified with the RNeasy Mini Kit (Qiagen, GmBH,

Germany) and hybridized to Agilent Human lncRNA 4×180K array slides at 65°C with rotation at 10 rpm for 17 h. The slides were scanned by an Agilent Microarray Scanner (G2565CA, Agilent Technologies). Data were extracted with Feature Extraction software, version 10.7 (Agilent Technologies), and the raw data analysis was performed with Gene Spring software, version 11.0 (Silicon Genetics, Redwood City, CA, USA).

Cell fractionation

Cells were washed with PBS, lysed in 500 μL of hypotonic buffer (10 mM HEPES (pH 7.9), 10 mM KCl, 0.1 mM EDTA, 0.1 mM EGTA, 0.15% NP-40) and protease inhibitors on ice for 15 min and centrifuged. The supernatant (cytoplasmic fraction) was collected. The nuclei-containing pellets were washed three times with 1 mL of hypotonic buffer, resuspended in 150 μL of ELB buffer, sonicated and centrifuged. The supernatant was collected as the nuclear fraction.

Immunofluorescence

Cells were seeded on microscope coverslips and fixed with 4% paraformaldehyde for 10 min. The cells were then washed three times with PBS, blocked in blocking buffer (3% BSA and 0.2% Triton X-100 in PBS) and incubated with the appropriate primary antibodies overnight at 4°C. The cells were then washed with washing buffer (0.2% BSA and 0.05% Triton X-100 in PBS) and incubated with Alexa Fluor-conjugated secondary antibodies (Life Technologies) for 1 h. The cells were stained with 4′,6-diamidino-2-phenylindole (DAPI) (1 μg/mL) for 10 min, then mounted and imaged with an LSM 780 microscope (Carl Zeiss, Oberkochen, Germany).

Hematoxylin and eosin staining and immunohistochemistry

The xenograft or metastatic liver samples were fixed in 4% paraformaldehyde, embedded in paraffin and sectioned. All sections were deparaffinized in xylene and dehydrated with a graded ethanol series. For hematoxylin and eosin staining, the liver sections were stained with hematoxylin and eosin. For immunohistochemistry, immunoreactivity was then detected in the sections using an UltraSensitive S-P kit (MX Biotechnologies, Fuzhou, China). Tumor sections were incubated with the antibodies as indicated overnight at 4°C. Peroxidase-labeled polymer and substrate-chromogen were then employed to visualize the antibodies staining. The slides were then counterstained with hematoxylin, dehydrated and mounted.

Supplementary references

1 Zhan Y, Du X, Chen H, Liu J, Zhao B, Huang D *et al.* Cytosporone B is an agonist for nuclear orphan receptor Nur77. *Nat Chem Biol* 2008; **4**: 548–556.

2 Li X xue, Wang Z jing, Zheng Y, Guan Y feng, Yang P bo, Chen X *et al.* Nuclear Receptor Nur77 Facilitates Melanoma Cell Survival under Metabolic Stress by Protecting Fatty Acid Oxidation. *Mol Cell* 2018; **69**: 480-492.e7.

3 Bian XL, Chen HZ, Yang PB, Li YP, Zhang FN, Zhang JY *et al.* Nur77 suppresses hepatocellular carcinoma via switching glucose metabolism toward gluconeogenesis through attenuating phosphoenolpyruvate carboxykinase sumoylation. *Nat Commun* 2017; **8**: 1–14.

Figure S1.

A, Nur77 inhibits HCC cell proliferation. Nur77 or Nur77 2G was transfected into Huh7 or HepG2 cells, cell viability was then determined by MTT assays. **B**, WFDC21P expression does not correlate with Nur77 in non-liver cancer cells. The RNA levels of WFDC21P and Nur77 were detected in 10 non-liver cancer cells (MCF7, HeLa, SH-SY5Y, MV3, U251, A375, A549, SGC-7921, U2OS, SW620). Heat maps and correlation charts were shown. **C**, The CPC scores (top) and GeneID scores (bottom) for WFDC21P, Nur77, several well-known lncRNAs and protein coding genes surrounding the WFDC21P gene locus were analyzed. **D,** The PhyloCSF score map of all potential open coding frames of WFDC21P. The WFDC21P sequence was entered in the UCSC web server, and the PhyloCSF scores are shown as the colored bars under each line. **E,** The WFDC21P sequence was cloned into a eukaryotic expression vector with an N-terminal start codon with three expression patterns (upper left). Western blotting was performed to detect C-terminal Flag-tagged proteins in Huh7 and HepG2 cells (right). The RNA level of WFDC21P are shown (bottom left). **F,** Nur77 localizes in both the cytoplasm and the nucleus in HCC cells. The cytoplasmic and nuclear fractions of Huh7 and HepG2 cells were prepared, and the expression of Nur77 were detected. Tubulin and PARP were used as cytoplasmic and nuclear controls, respectively.

Figure S2.

A and **B,** The efficiency of WFDC21P overexpression in Figure 2A-B (**A**) and knockdown in Figure 2C-D (**B**) in Huh7 and HepG2 cells. Cells were collected and subjected to RT-qPCR to detect the WFDC21P expression level. **C,** WFDC21P cannot influence cell

survival in HCC cells. WFDC21P was overexpressed or knocked down in Huh7 or HepG2 cells as indicated. Cell death was determined by Annexin V/PI double staining and caspase 3 cleavage. Apoptotic inducer staurosporine (STS) (1 μM) was used as a positive control. **D,** The efficiency of WFDC21P knockdown and Nur77 overexpression in Huh7 and HepG2 cells in Figure 2E-F. Cells were collected and subjected to RT-qPCR or western blotting to detect the WFDC21P expression level or Nur77 expression level. **E,** A schematic diagram of the MS2 and MS2bs system. HA-2 × MS2 interacts with the stem loop structure of MS2bs, which is transcribed after WFDC21P under the control of the cytomegalovirus (CMV) promoter. Proteins that interact with WFDC21P are pulled down by immunoprecipitation using the anti-HA antibody. **F**, WFDC21P does not interact with PEPCK1. The interactions between WFDC21P and PEPCK1 were determined by protein immunoprecipitation using the MS2/MS2bs system (left) or RIP (right). The expression of WFDC21P and the efficiency of immunoprecipitation were determined by RT-qPCR and western blotting respectively. **G**, PEPCK1 cannot influence WFDC21P expression in HCC cells. PEPCK1 was overexpressed or knocked down in Huh7 or HepG2 cells. The expressions of WFDC21P and PEPCK1 were detected. **H**, WFDC21P does not influence PEPCK1 expression in HCC cells. WFDC21P was overexpressed or knocked down in Huh7 or HepG2 cells as shown in Supplementary Fig. 2A-B. The expressions of PEPCK1 were detected. **I**, WFDC21P cannot influence cell apoptosis in xenograft tissues. Apoptosis was determined by immunohistochemically staining of activate caspase 3 in xenograft tissues from Figure 2G and 2H. **J,** The efficiency of WFDC21P overexpression (left) and knockdown (right) in Huh7-derived xenograft tumors from Figure 2G and 2H.

Figure S3.

A and **B,** The efficiency of WFDC21P overexpression (**A**) and knockdown (**B**) in Huh7 cells used in Figure 3.

Figure S4.

A, WFDC21P is lowly expressed in HCC samples. WFDC21P expression levels in 39 para-carcinoma samples and 116 HCC samples were detected by RT-qPCR. **B,** The expressions of Nur77 in clinical HCC tissues and their paired para-carcinoma tissues (n=18) were detected by RT-qPCR. **C,** Nur77 expression levels in carcinoma is decreased compared to para-carcinoma. Nur77 expression levels in 18 normal liver samples and 25 HCC samples were shown. **D,** Nur77 expression levels are decreased as HCC progress. Nur77 expression levels in 18 normal liver samples and 11 HCC samples grouped into stages II-III were shown.

Figure S5.

A-B, Nur77 inhibits glycolysis in HCC cells. Nur77 were overexpressed (**A**) or knocked down (**B**) in Huh7 or HepG2 cells. Glucose uptake and lactate production were then detected. **C-D,** The efficiency of WFDC21P overexpression (**C**) and knockdown (**D**) in Huh7 and HepG2 cells used in Figure 5B-D. **E,** PFKP is the main isoform expressed in Huh7 and HepG2 cells. The expression levels of PFKP, PFKM and PFKL in Huh7 and HepG2 cells were determined by RT-qPCR. **F-G,** WFDC21P does not obviously interact with HK2 or GAPDH. The interactions between WFDC21P and HK2 (**F**) or GAPDH (**G**) were determined by protein immunoprecipitation using the MS2/MS2bs system or RIP. β-

actin was used as a normalizing control. **H-I**, Mapping the interaction domain of WFDC21P with PFKP (**H**) and PKM2 (**I**). Different deletion mutants of WFDC21P were co-transfected with PFKP or PKM2 into Huh7 cells. The interactions between WFDC21P deletions with PFKP and PKM2 were determined by protein immunoprecipitation using the MS2/MS2bs system.

Figure S6.

A and **B,** The efficiency of WFDC21P overexpression (**A**) and knockdown (**B**) in Huh7 and HepG2 cells used in Figure 6. **C** and **D**, WFDC21P does not affect GAPDH activity. The GAPDH activity was measured in WFDC21P overexpression (**C**) or knockdown (**D**) Huh7 and HepG2 cells from Supplementary Figure S6A-B. **E,** WFDC21P-ΔE3 does not affect PFK1 activity in Huh7 and HepG2 cells. The expression levels of WFDC21P and its mutant were determined by RT-qPCR (right). **F-G**, The inhibitory effects of WFDC21P-ΔE3 on glycolysis (**F**) and cell proliferation (**G**) are attenuated. **H**, The expression level of WFDC21P was measured in the PFKP polymer assay. Huh7 and HepG2 cells that transfected with WFDC21P and Flag-PFKP were subjected to RT-qPCR. **I**, WFDC21P-ΔE2 does not affect PK activity in Huh7 and HepG2 cells (top), and indicated RNA expression levels were determined by RT-qPCR (bottom). **J-K**, The inhibitory effects of WFDC21P-ΔE2 on glycolysis (**J**) and cell proliferation (**K**) are attenuated.

Figure S7.

A-B, Nur77 inhibits HCC cell growth through glycolysis regulation. Nur77 was overexpressed (**A**) or knocked down (**B**) in Huh7 or HepG2 cells. The cell viability was

then determined in the presence or absence of 2-DG (5 mM). **C**, The knockdown efficiency of WFDC21P and PFKP in Huh7 cells. Cells were collected and subjected to RT-qPCR to detect the WFDC21P (left) or PFKP (right) expression levels. **D,** The knockdown efficiency of WFDC21P and PKM2 in Huh7 cells. Cells were collected and subjected to RT-qPCR to detect the WFDC21P (left) or PKM2 (right) expression levels.

Supplementary table 1. Relationships between WFDC21P expression and the clinicopathological features of patients with HCC.

Supplementary table 2. Detailed clinical information of HCC patients used in this study.

Supplementary table 3. The list of WFDC21P-interacting proteins identified by mass spectrometry.

Supplementary Figure 1

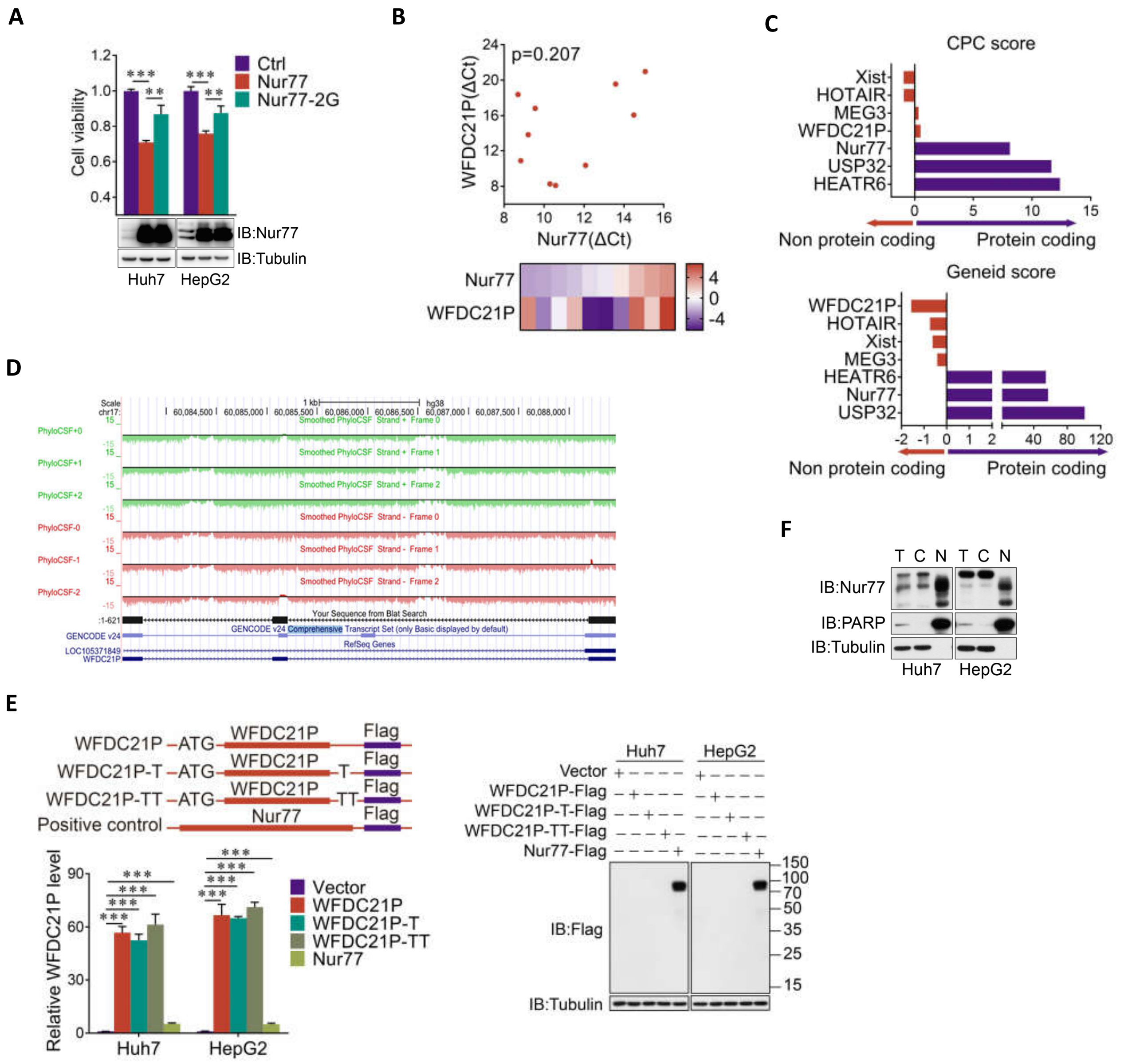

Supplementary Figure 2

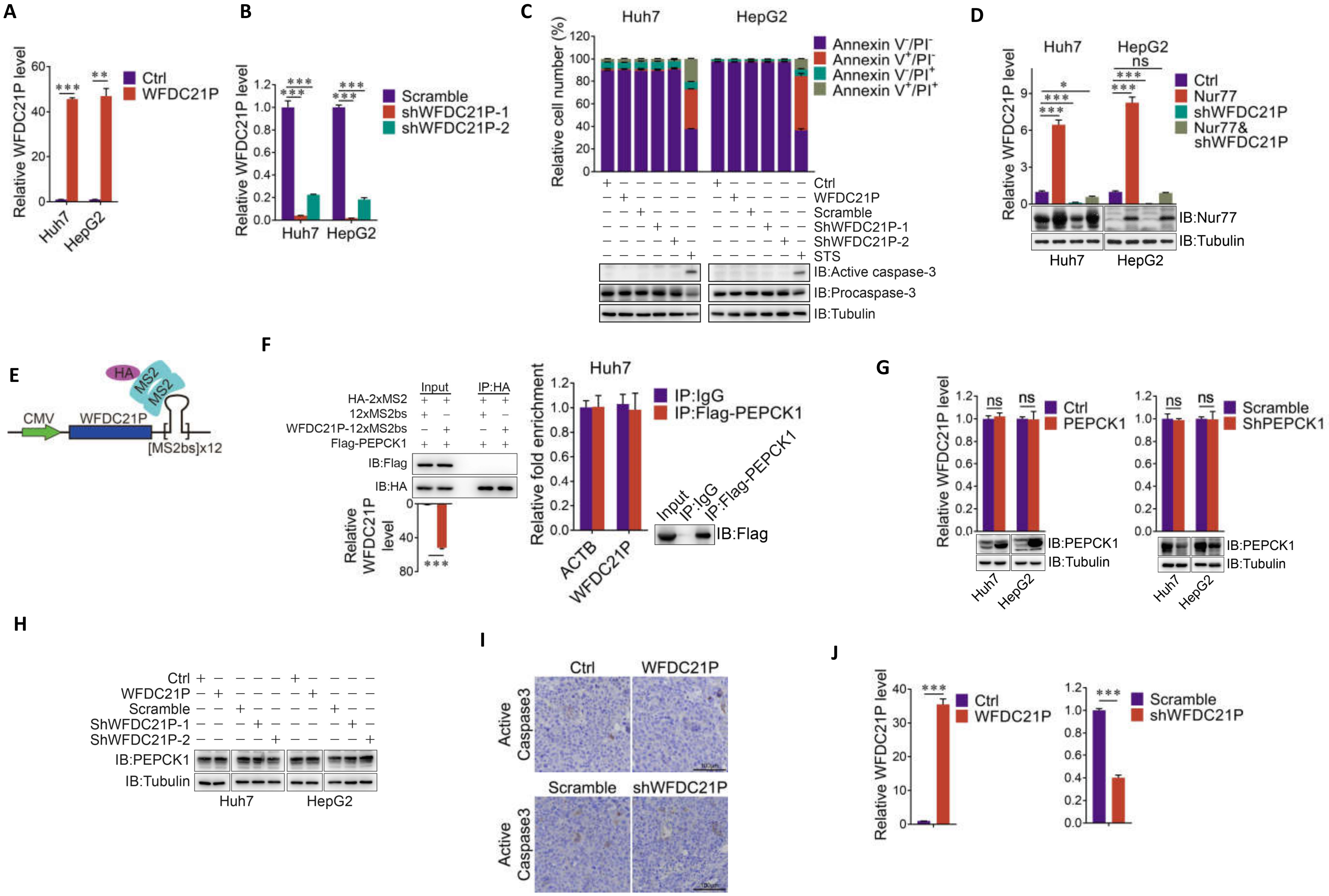

Supplementary Figure 3

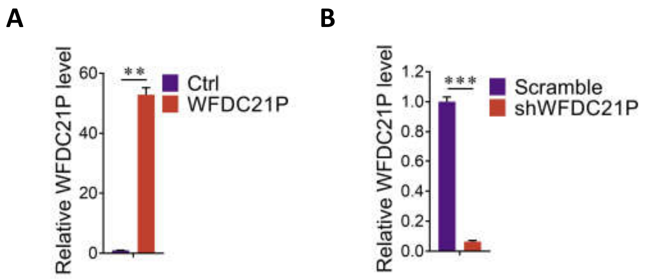

Supplementary Figure 4

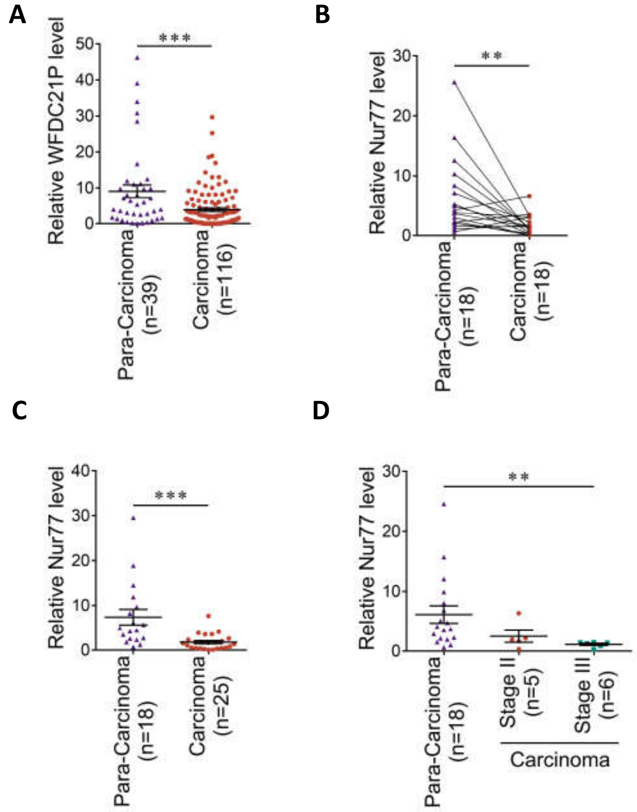

Supplementary Figure 5

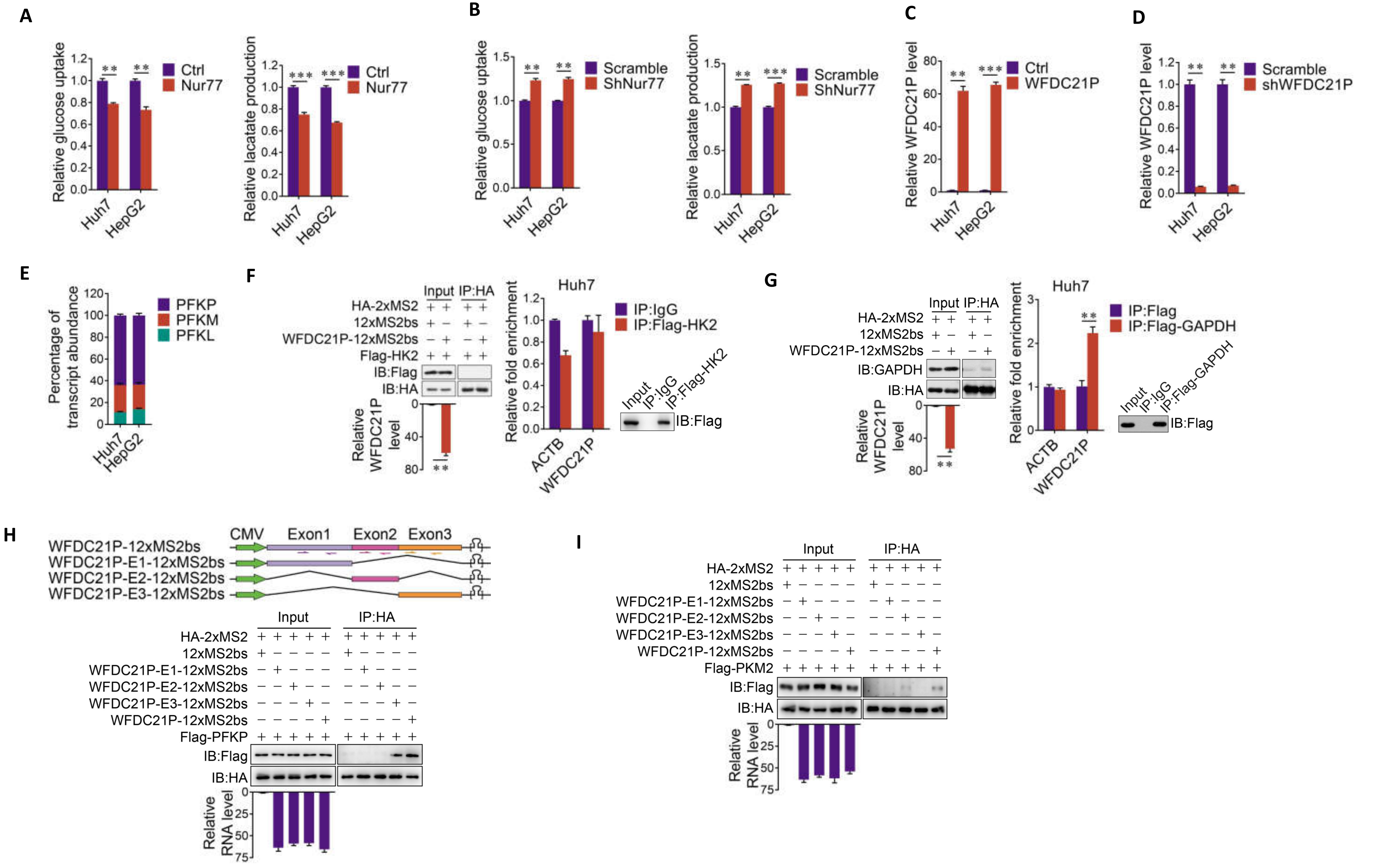

Supplementary Figure 6

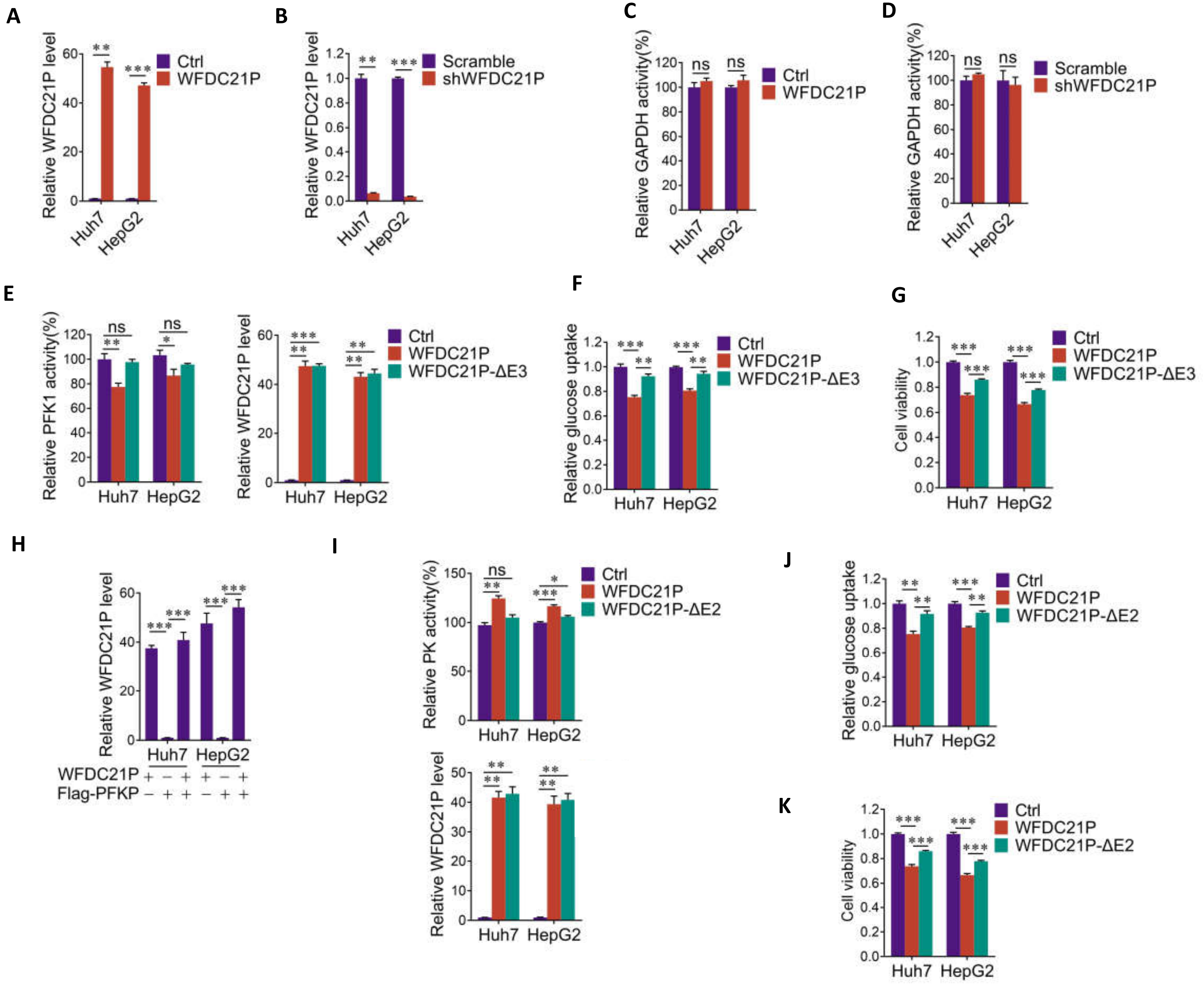

Supplementary Figure 7

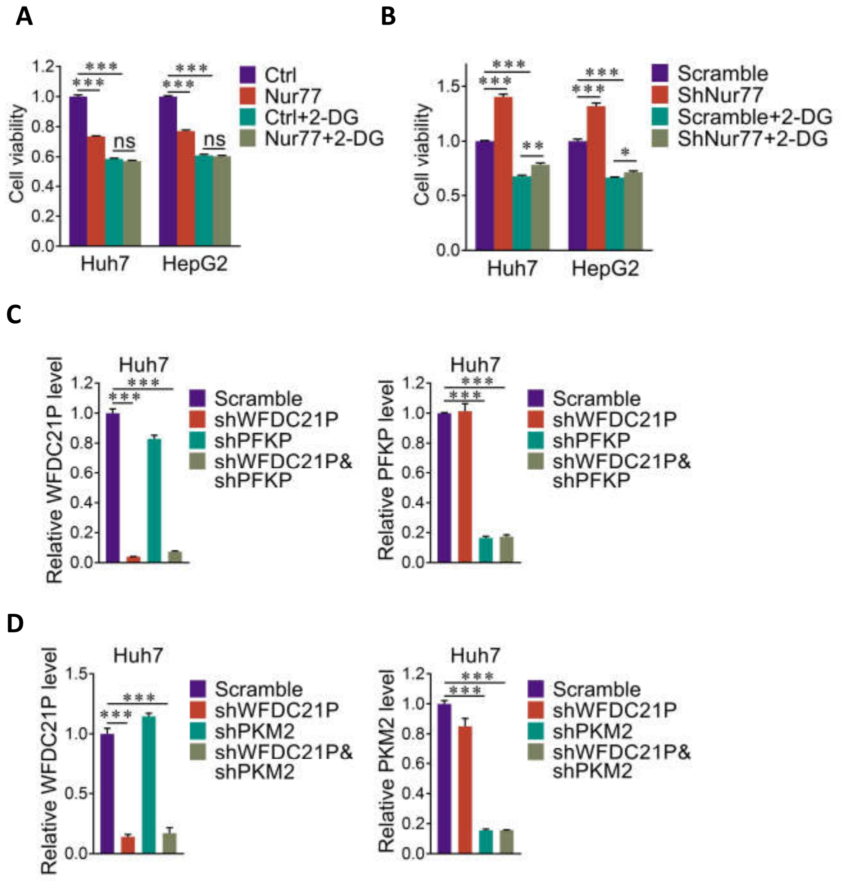

Supplement: Supplementary file 1 — Supplementary information [file 41388_2020_1158_MOESM1_ESM.pdf]
