# Supplementary material for: Nur77-activated lncRNA WFDC21P attenuates hepatocarcinogenesis via modulating glycolysis
Source: Oncogene. 2020 Jan 20;39(11):2408–23. doi: 10.1038/s41388-020-1158-y (PMC7067692; doi:10.1038/s41388-020-1158-y)
Supplement: Supplementary file 2 — Supplementary Table 1 [file 41388_2020_1158_MOESM2_ESM.pdf]

Table S1. Relationships between WFDC21P expression and the clinicopathological features of patients with HCC.

feature	No. of patients	WFDC21P		χ2	*p* -value
		High	Low		
Gender				3.31	0.0688
Male	102	33	69		
Female	14	8	6		
Age (years)				0.0544	0.8139
⩽60	78	27	51		
>60	38	14	24		
Tumorous number				7.165	0.0074
1	70	28	42		
⩾2	17	1	16		
unknown	29				
Tumor size (cm)				8.488	0.0036
⩽5	58	28	30		
>5	58	13	45		
Embolus				6.178	0.0129
No	25	9	16		
Yes	20	1	19		
unknown	71				
Tumor capsule				0.7	0.4028
No	11	4	7		
Yes	34	8	26		
unknown	71				
AJCC stage				4.846	0.0277
1-2	42	17	25		
3-4	13	1	12		
unknown	61				
TNM				4.447	0.035
T1-2	44	17	27		
T3-4	13	1	12		
unknown	59				
Survival				4.724	0.0297
survival	49	25	24		
deceased	42	12	30		
unknown	25				
