# Supplementary material for: Nur77-activated lncRNA WFDC21P attenuates hepatocarcinogenesis via modulating glycolysis
Source: Oncogene. 2020 Jan 20;39(11):2408–23. doi: 10.1038/s41388-020-1158-y (PMC7067692; doi:10.1038/s41388-020-1158-y)
Supplement: Supplementary file 3 — Supplementary Table 2 [file 41388_2020_1158_MOESM3_ESM.pdf]

Table S2. Detailed clinical information of HCC patients used in this study.

Number	Organ	Pathology Type	Histologic type	Gender	Age	AJCC Stage	Tumor capsule	Tumor size (cm)	Tumor number	Tumor embolus	Primary organ	T	N	M	Surgery Date	Visit Date	Survival Time	Survival Status
1	Liver	Hepatocarcinoma	Carcinoma	Male	12	*N/A*	*N/A*	4	1	Yes	Yes	*N/A*	*N/A*	*N/A*	2008/8/8	2015.9	85	survival
2	Liver	Hepatocarcinoma	Para/Carcinoma	Female	23	*N/A*	*N/A*	9	*N/A*	*N/A*	Yes	*N/A*	N0	M0	*N/A*	*N/A*	*N/A*	*N/A*
3	Liver	Hepatocarcinoma	Carcinoma	Male	29	*N/A*	*N/A*	8	*N/A*	*N/A*	Yes	*N/A*	*N/A*	*N/A*	2006/4/20	2015.9	113	survival
4	Liver	Hepatocarcinoma	Carcinoma	Male	37	2	incomplete	4.5	2	*N/A*	Yes	T2	N0	M0	2012/5/4	2015/9/5	40	deceased
7	Liver	Hepatocarcinoma	Carcinoma	Male	38	*N/A*	*N/A*	2.2	1	Yes	Yes	*N/A*	*N/A*	*N/A*	2008/7/29	2015.9	86	survival
5	Liver	Hepatocarcinoma	Carcinoma	Male	38	2	complete	1	2	*N/A*	Yes	T2	N0	M0	2012/1/18	2016.05	51	survival
6	Liver	Hepatocarcinoma	Carcinoma	Male	38	1	complete	3	1	*N/A*	Yes	T1	N0	M0	2012/2/10	2014/9/1	29	deceased
10	Liver	Hepatocarcinoma	Para/Carcinoma	Male	39	*N/A*	*N/A*	14	2	No	Yes	*N/A*	*N/A*	*N/A*	2008/6/11	2015.9	87	survival
8	Liver	Hepatocarcinoma	Para/Carcinoma	Male	39	*N/A*	*N/A*	8.5	1	No	Yes	*N/A*	*N/A*	*N/A*	2009/8/25	2015.9	73	survival
9	Liver	Hepatocarcinoma	Carcinoma	Male	39	*N/A*	*N/A*	7	1	No	Yes	*N/A*	*N/A*	*N/A*	2009/9/7	2015.9	72	survival
11	Liver	Hepatocarcinoma	Carcinoma	Male	40	1	complete	4.5	1	*N/A*	Yes	T1	N0	M0	2012/2/21	2016.05	50	survival
13	Liver	Hepatocarcinoma	Carcinoma	Male	41	*N/A*	*N/A*	3	*N/A*	No	Yes	*N/A*	N0	M0	*N/A*	*N/A*	*N/A*	*N/A*
14	Liver	Hepatocarcinoma	Para/Carcinoma	Male	41	*N/A*	*N/A*	5	*N/A*	*N/A*	Yes	*N/A*	N0	M0	*N/A*	*N/A*	*N/A*	*N/A*
12	Liver	Hepatocarcinoma	Para/Carcinoma	Male	41	1	*N/A*	9	1	*N/A*	Yes	T1	N0	M0	2012/4/28	2016.05	48	survival
17	Liver	Hepatocarcinoma	Carcinoma	Male	42	2	*N/A*	5	*N/A*	*N/A*	Yes	T2	N0	M0	*N/A*	*N/A*	*N/A*	*N/A*
18	Liver	Hepatocarcinoma	Carcinoma	Male	42	*N/A*	incomplete	7.1	*N/A*	*N/A*	Yes	*N/A*	N0	M0	*N/A*	*N/A*	*N/A*	*N/A*
15	Liver	Hepatocarcinoma	Para/Carcinoma	Male	42	1	complete	7	1	*N/A*	Yes	T1	N0	M0	2012/2/3	2016.05	50	survival
16	Liver	Hepatocarcinoma	Para/Carcinoma	Male	42	1	complete	4	1	*N/A*	Yes	T1	N0	M0	2012/3/1	2016.05	49	survival
21	Liver	Hepatocarcinoma	Carcinoma	Male	43	*N/A*	*N/A*	5.5	*N/A*	*N/A*	Yes	*N/A*	N0	M0	*N/A*	*N/A*	*N/A*	*N/A*
20	Liver	Hepatocarcinoma	Carcinoma	Male	43	1	complete	10	1	*N/A*	Yes	T1	N0	M0	2012/2/15	2016.05	50	survival
19	Liver	Hepatocarcinoma	Para/Carcinoma	Male	43	1	incomplete	1.8	1	*N/A*	Yes	T1	N0	M0	2012/4/21	2016.05	48	survival
22	Liver	Hepatocarcinoma	Para/Carcinoma	Male	44	*N/A*	*N/A*	6	1	No	Yes	*N/A*	*N/A*	*N/A*	2008/12/10	2011/3/9	27	deceased
27	Liver	Hepatocarcinoma	Carcinoma	Male	45	*N/A*	*N/A*	3	1	Yes	Yes	*N/A*	*N/A*	*N/A*	2009/7/7	2015.9	74	survival
25	Liver	Hepatocarcinoma	Para/Carcinoma	Male	45	*N/A*	*N/A*	4	1	Yes	Yes	*N/A*	*N/A*	*N/A*	2010/12/3	2012/4/15	16	deceased
23	Liver	Hepatocarcinoma	Para/Carcinoma	Male	45	1	complete	16	1	*N/A*	Yes	T1	N0	M0	2012/2/17	2016/6/14	51	deceased
24	Liver	Hepatocarcinoma	Para/Carcinoma	Male	45	*N/A*	*N/A*	9	1	No	Yes	*N/A*	*N/A*	*N/A*	2009/5/21	2012/2/21	33	deceased
28	Liver	Hepatocarcinoma	Carcinoma	Male	45	*N/A*	*N/A*	7	1	No	Yes	*N/A*	*N/A*	*N/A*	2013/1/25	2014/8/31	19	deceased
26	Liver	Hepatocarcinoma	Carcinoma	Male	45	*N/A*	*N/A*	5.5	1	Yes	Yes	*N/A*	*N/A*	*N/A*	2013/5/16	2015/6/16	25	deceased
29	Liver	Hepatocarcinoma	Carcinoma	Male	46	*N/A*	*N/A*	3.5	1	Yes	Yes	*N/A*	*N/A*	*N/A*	2012/1/11	2014/11/7	34	deceased
33	Liver	Hepatocarcinoma	Carcinoma	Male	47	*N/A*	*N/A*	10	1	No	Yes	*N/A*	*N/A*	*N/A*	2011/4/20	2015.9	53	survival
32	Liver	Hepatocarcinoma	Para/Carcinoma	Male	47	*N/A*	*N/A*	7.5	1	No	Yes	*N/A*	*N/A*	*N/A*	2010/3/12	2012/7/26	28	deceased
30	Liver	Hepatocarcinoma	Carcinoma	Male	47	1	complete	6	1	*N/A*	Yes	T1	N0	M0	2012/2/29	2013/10/15	20	deceased
31	Liver	Hepatocarcinoma	Carcinoma	Male	47	*N/A*	complete	13.2	*N/A*	*N/A*	Yes	*N/A*	N0	M0	*N/A*	*N/A*	*N/A*	*N/A*
35	Liver	Hepatocarcinoma	Carcinoma	Male	48	2	incomplete	3.5	7	*N/A*	Yes	T2	N0	M0	2012/2/6	2015/3/15	37	deceased
36	Liver	Hepatocarcinoma	Carcinoma	Female	48	*N/A*	*N/A*	3.5	1	Yes	Yes	*N/A*	*N/A*	*N/A*	2011/2/14	2015.9	55	survival
34	Liver	Hepatocarcinoma	Para/Carcinoma	Male	48	3	incomplete	6	3	*N/A*	Yes	T3	N0	M0	2012/1/18	2016.05	51	survival
38	Liver	Hepatocarcinoma	Para/Carcinoma	Male	49	*N/A*	*N/A*	11	1	No	Yes	*N/A*	*N/A*	*N/A*	2008/5/13	2008/12/9	7	deceased
37	Liver	Hepatocarcinoma	Carcinoma	Male	49	*N/A*	*N/A*	9	1	No	Yes	*N/A*	*N/A*	*N/A*	2009/7/3	2010/7/30	12	deceased
40	Liver	Hepatocarcinoma	Carcinoma	Male	50	3	incomplete	7	7	*N/A*	Yes	T3	N0	M0	2012/5/8	2015/2/15	33	deceased
42	Liver	Hepatocarcinoma	Carcinoma	Male	50	*N/A*	*N/A*	5	1	Yes	Yes	*N/A*	*N/A*	*N/A*	2009/11/16	2015/1/7	62	deceased
39	Liver	Hepatocarcinoma	Para/Carcinoma	Female	50	1	complete	2.5	1	*N/A*	Yes	T1	N0	M0	2012/1/18	2016.05	51	survival
41	Liver	Hepatocarcinoma	Carcinoma	Female	50	2	*N/A*	10	*N/A*	*N/A*	Yes	T2	N0	M0	*N/A*	*N/A*	*N/A*	*N/A*
45	Liver	Hepatocarcinoma	Para/Carcinoma	Male	51	*N/A*	*N/A*	2.4	1	Yes	Yes	*N/A*	*N/A*	*N/A*	2008/7/23	2009/5/2	10	deceased
43	Liver	Hepatocarcinoma	Para/Carcinoma	Male	51	*N/A*	*N/A*	7	1	No	Yes	*N/A*	*N/A*	*N/A*	2009/9/23	2015.9	72	survival

44	Liver	Hepatocarcinoma	Carcinoma	Male	51	*N/A*	*N/A*	5	1	Yes	Yes	*N/A*	*N/A*	*N/A*	2010/1/14	2012/8/20	31	deceased
46	Liver	Hepatocarcinoma	Carcinoma	Male	52	*N/A*	*N/A*	5	1	Yes	Yes	*N/A*	*N/A*	*N/A*	2008/9/9	2015.9	84	survival
52	Liver	Hepatocarcinoma	Para/Carcinoma	Male	53	*N/A*	*N/A*	6	>2	No	Yes	*N/A*	*N/A*	*N/A*	2006/10/17	2007/7/26	9	deceased
51	Liver	Hepatocarcinoma	Carcinoma	Male	53	*N/A*	*N/A*	17	1	No	Yes	*N/A*	*N/A*	*N/A*	2009/6/30	2011/9/16	21	deceased
47	Liver	Hepatocarcinoma	Para/Carcinoma	Male	53	3	*N/A*	5.5	*N/A*	*N/A*	Yes	T3	N0	M0	*N/A*	*N/A*	*N/A*	*N/A*
48	Liver	Hepatocarcinoma	Para/Carcinoma	Male	53	3	*N/A*	7	*N/A*	*N/A*	Yes	T3	N0	M0	*N/A*	*N/A*	*N/A*	*N/A*
50	Liver	Hepatocarcinoma	Para/Carcinoma	Male	53	*N/A*	*N/A*	6	1	No	Yes	*N/A*	*N/A*	*N/A*	2008/12/9	2012/2/2	38	deceased
49	Liver	Hepatocarcinoma	Carcinoma	Male	53	2	*N/A*	10	*N/A*	*N/A*	Yes	T2	N0	M0	*N/A*	*N/A*	*N/A*	*N/A*
53	Liver	Hepatocarcinoma	Carcinoma	Male	54	1	complete	3.8	1	*N/A*	Yes	T1	N0	M0	2012/2/2	2016.05	50	survival
54	Liver	Hepatocarcinoma	Para/Carcinoma	Female	54	4	*N/A*	13	*N/A*	*N/A*	Yes	T4	N0	M1	*N/A*	*N/A*	*N/A*	*N/A*
55	Liver	Hepatocarcinoma	Para/Carcinoma	Female	54	*N/A*	*N/A*	10	2	No	Yes	*N/A*	*N/A*	*N/A*	2007/10/10	2009/4/4	18	deceased
59	Liver	Hepatocarcinoma	Carcinoma	Male	55	*N/A*	*N/A*	10	1	No	Yes	*N/A*	*N/A*	*N/A*	2007/3/2	2008/5/1	14	deceased
60	Liver	Hepatocarcinoma	Carcinoma	Male	55	*N/A*	*N/A*	5	1	Yes	Yes	*N/A*	*N/A*	*N/A*	2011/2/22	2012/6/22	16	deceased
57	Liver	Hepatocarcinoma	Carcinoma	Male	55	3	complete	7	3	*N/A*	Yes	T3	N0	M0	2012/2/9	2012/8/9	18	deceased
56	Liver	Hepatocarcinoma	Carcinoma	Male	55	1	complete	12.5	1	*N/A*	Yes	T1	N0	M0	2012/4/20	2016.05	48	survival
58	Liver	Hepatocarcinoma	Carcinoma	Male	55	2	*N/A*	6	*N/A*	*N/A*	Yes	T2	N0	M0	*N/A*	*N/A*	*N/A*	*N/A*
61	Liver	Hepatocarcinoma	Carcinoma	Male	56	*N/A*	complete	3.3	1	*N/A*	Yes	T1	N0	M1	2011/3/21	2014/9/20	42	deceased
62	Liver	Hepatocarcinoma	Carcinoma	Male	56	2	incomplete	1.6	2	*N/A*	Yes	T2	N0	M0	2011/3/25	2016.05	61	survival
63	Liver	Hepatocarcinoma	Carcinoma	Male	56	2	complete	4	3	*N/A*	Yes	T2	N0	M0	2012/4/21	2016.05	48	survival
64	Liver	Hepatocarcinoma	Carcinoma	Male	56	2	complete	5	2	*N/A*	Yes	T2	N0	M0	2012/4/28	2015/12/4	44	deceased
65	Liver	Hepatocarcinoma	Carcinoma	Male	57	1	complete	1.5	1	*N/A*	Yes	T1	N0	M0	2012/4/19	2014/12/3	32	deceased
68	Liver	Hepatocarcinoma	Carcinoma	Male	58	*N/A*	incomplete	3.5	1	*N/A*	Yes	T1	N1	M0	2012/4/25	2013/12/15	20	deceased
69	Liver	Hepatocarcinoma	Carcinoma	Female	58	*N/A*	*N/A*	4.7	*N/A*	*N/A*	Yes	*N/A*	*N/A*	*N/A*	2009/12/2	2015.9	69	survival
66	Liver	Hepatocarcinoma	Carcinoma	Male	58	2	complete	4.5	2	*N/A*	Yes	T2	N0	M0	2012/2/1	2016.05	50	survival
67	Liver	Hepatocarcinoma	Para/Carcinoma	Male	58	3	complete	12	4	*N/A*	Yes	T3	N0	M0	2012/2/8	2013/8/15	18	deceased
70	Liver	Hepatocarcinoma	Para/Carcinoma	Male	59	*N/A*	*N/A*	4.5	*N/A*	*N/A*	Yes	*N/A*	N0	M0	*N/A*	*N/A*	*N/A*	*N/A*
76	Liver	Hepatocarcinoma	Carcinoma	Male	60	*N/A*	*N/A*	3.5	1	Yes	Yes	*N/A*	*N/A*	*N/A*	2010/1/18	2015.9	68	survival
72	Liver	Hepatocarcinoma	Carcinoma	Female	60	1	complete	3	1	*N/A*	Yes	T1	N0	M0	2011/4/7	2016.05	60	survival
71	Liver	Hepatocarcinoma	Carcinoma	Male	60	1	complete	6	1	*N/A*	Yes	T1	N0	M0	2012/4/28	2016.05	48	survival
77	Liver	Hepatocarcinoma	Para/Carcinoma	Male	60	*N/A*	*N/A*	4.5	1	Yes	Yes	*N/A*	*N/A*	*N/A*	2007/4/26	2015.9	101	survival
78	Liver	Hepatocarcinoma	Para/Carcinoma	Male	60	*N/A*	*N/A*	4.5	1	Yes	Yes	*N/A*	*N/A*	*N/A*	2007/7/19	2015.9	98	survival
75	Liver	Hepatocarcinoma	Para/Carcinoma	Male	60	*N/A*	*N/A*	7	1	No	Yes	*N/A*	*N/A*	*N/A*	2009/8/10	2015.9	73	survival
73	Liver	Hepatocarcinoma	Carcinoma	Male	60	1	complete	3.5	1	*N/A*	Yes	T1	N0	M0	2012/5/7	2013/11/15	18	deceased
74	Liver	Hepatocarcinoma	Carcinoma	Female	60	*N/A*	*N/A*	4	*N/A*	*N/A*	Yes	*N/A*	N0	M0	*N/A*	*N/A*	*N/A*	*N/A*
79	Liver	Hepatocarcinoma	Carcinoma	Female	61	1	complete	2.5	1	*N/A*	Yes	T1	N0	M0	2012/1/18	2016.05	51	survival
80	Liver	Hepatocarcinoma	Carcinoma	Male	61	2	complete	3.5	2	*N/A*	Yes	T2	N0	M0	2012/4/26	2016.05	48	survival
82	Liver	Hepatocarcinoma	Para/Carcinoma	Male	61	*N/A*	*N/A*	10	1	No	Yes	*N/A*	*N/A*	*N/A*	2009/8/12	2014/6/2	48	deceased
83	Liver	Hepatocarcinoma	Para/Carcinoma	Male	61	*N/A*	*N/A*	5	2	No	Yes	*N/A*	*N/A*	*N/A*	2011/7/1	2015.9	50	survival
81	Liver	Hepatocarcinoma	Carcinoma	Male	61	3	*N/A*	6	*N/A*	*N/A*	Yes	T3	N0	M0	*N/A*	*N/A*	*N/A*	*N/A*
87	Liver	Hepatocarcinoma	Carcinoma	Male	62	2	*N/A*	7	*N/A*	*N/A*	Yes	T2	N0	M0	*N/A*	*N/A*	*N/A*	*N/A*
89	Liver	Hepatocarcinoma	Carcinoma	Male	62	*N/A*	*N/A*	6	*N/A*	*N/A*	Yes	*N/A*	N0	M0	*N/A*	*N/A*	*N/A*	*N/A*
84	Liver	Hepatocarcinoma	Carcinoma	Male	62	1	incomplete	5	1	*N/A*	Yes	T1	N0	M0	2012/1/5	2016.05	51	survival
85	Liver	Hepatocarcinoma	Para/Carcinoma	Male	62	3	complete	7	2	*N/A*	Yes	T3	N0	M0	2012/1/18	2016.05	51	survival
86	Liver	Hepatocarcinoma	Carcinoma	Male	62	1	complete	2	1	*N/A*	Yes	T1	N0	M0	2012/2/21	2016.06	50	survival
88	Liver	Hepatocarcinoma	Para/Carcinoma	Male	62	*N/A*	*N/A*	4	*N/A*	*N/A*	Yes	*N/A*	N0	M0	*N/A*	*N/A*	*N/A*	*N/A*
93	Liver	Hepatocarcinoma	Carcinoma	Male	63	*N/A*	*N/A*	3.5	1	Yes	Yes	*N/A*	*N/A*	*N/A*	2009/6/22	2011/12/5	30	deceased
91	Liver	Hepatocarcinoma	Carcinoma	Male	63	3	*N/A*	10.5	1	*N/A*	Yes	T3	N0	M0	2010/12/31	2011/11/15	11	deceased
90	Liver	Hepatocarcinoma	Carcinoma	Male	63	1	complete	8	1	*N/A*	Yes	T1	N0	M0	2012/4/11	2016/7/7	49	deceased
92	Liver	Hepatocarcinoma	Carcinoma	Male	63	1	complete	6	1	*N/A*	Yes	T1	N0	M0	2012/9/5	2016.06	43	survival

94	Liver	Hepatocarcinoma	Para/Carcinoma	Male	64	*N/A*	*N/A*	4.5	1	Yes	Yes	*N/A*	*N/A*	*N/A*	2011/2/23	2015.9	55	survival
95	Liver	Hepatocarcinoma	Carcinoma	Male	65	1	complete	6	1	*N/A*	Yes	T1	N0	M0	2010/12/29	2016.04	64	survival
97	Liver	Hepatocarcinoma	Para/Carcinoma	Male	65	*N/A*	*N/A*	4	1	Yes	Yes	*N/A*	*N/A*	*N/A*	2009/10/27	2015.9	71	survival
96	Liver	Hepatocarcinoma	Carcinoma	Female	65	3	*N/A*	7	*N/A*	*N/A*	Yes	T3	N0	M0	*N/A*	*N/A*	*N/A*	*N/A*
99	Liver	Hepatocarcinoma	Para/Carcinoma	Male	66	*N/A*	*N/A*	2.5	1	Yes	Yes	*N/A*	*N/A*	*N/A*	2007/2/5	2015.9	103	survival
100	Liver	Hepatocarcinoma	Para/Carcinoma	Female	66	*N/A*	*N/A*	5	*N/A*	*N/A*	Yes	*N/A*	*N/A*	*N/A*	2008/8/1	2011/1/18	29	deceased
98	Liver	Hepatocarcinoma	Carcinoma	Male	66	*N/A*	*N/A*	1.5	*N/A*	No	Yes	*N/A*	N0	M0	*N/A*	*N/A*	*N/A*	*N/A*
101	Liver	Hepatocarcinoma	Carcinoma	Male	67	1	complete	9	1	*N/A*	Yes	T1	N0	M0	2011/4/13	2016.05	60	survival
104	Liver	Hepatocarcinoma	Carcinoma	Male	68	*N/A*	*N/A*	3	*N/A*	*N/A*	Yes	*N/A*	*N/A*	*N/A*	2009/8/26	2015.9	73	survival
102	Liver	Hepatocarcinoma	Carcinoma	Male	68	1	incomplete	3	1	*N/A*	Yes	T1	N0	M0	2012/4/11	2014/9/1	29	deceased
103	Liver	Hepatocarcinoma	Carcinoma	Male	68	*N/A*	*N/A*	7	*N/A*	*N/A*	Yes	*N/A*	N0	M0	*N/A*	*N/A*	*N/A*	*N/A*
106	Liver	Hepatocarcinoma	Carcinoma	Female	69	3	*N/A*	7	*N/A*	*N/A*	Yes	T3	N0	M0	*N/A*	*N/A*	*N/A*	*N/A*
105	Liver	Hepatocarcinoma	Carcinoma	Male	69	1	complete	3	1	*N/A*	Yes	T1	N0	M0	2012/4/7	2016.05	48	survival
107	Liver	Hepatocarcinoma	Para/Carcinoma	Male	69	*N/A*	*N/A*	10	1	No	Yes	*N/A*	*N/A*	*N/A*	2012/11/27	2015/1/4	26	deceased
109	Liver	Hepatocarcinoma	Carcinoma	Male	70	1	complete	5	1	*N/A*	Yes	T1	N0	M0	2011/1/6	2014/4/15	39	deceased
108	Liver	Hepatocarcinoma	Carcinoma	Male	70	1	complete	4.5	1	*N/A*	Yes	T1	N0	M0	2012/2/15	2016.05	50	survival
110	Liver	Hepatocarcinoma	Carcinoma	Male	70	3	*N/A*	6	*N/A*	*N/A*	Yes	T3	N0	M0	*N/A*	*N/A*	*N/A*	*N/A*
111	Liver	Hepatocarcinoma	Carcinoma	Female	71	1	incomplete	4	1	*N/A*	Yes	T1	N0	M0	2012/2/15	2016.05	50	survival
112	Liver	Hepatocarcinoma	Carcinoma	Male	72	*N/A*	*N/A*	6	1	No	Yes	*N/A*	*N/A*	*N/A*	2007/6/4	2013/4/7	70	deceased
113	Liver	Hepatocarcinoma	Carcinoma	Male	72	*N/A*	*N/A*	4	1	Yes	Yes	*N/A*	*N/A*	*N/A*	2010/10/14	2013/10/6	36	deceased
114	Liver	Hepatocarcinoma	Carcinoma	Male	73	*N/A*	*N/A*	2.5	*N/A*	No	Yes	*N/A*	N0	M0	*N/A*	*N/A*	*N/A*	*N/A*
115	Liver	Hepatocarcinoma	Para/Carcinoma	Male	74	*N/A*	*N/A*	6	1	No	Yes	*N/A*	*N/A*	*N/A*	2008/12/8	2015.9	81	survival
116	Liver	Hepatocarcinoma	Carcinoma	Male	79	1	complete	7.5	1	*N/A*	Yes	T1	N0	M0	2012/4/17	2013/7/1	15	deceased
