# Supplementary material for: Nur77-activated lncRNA WFDC21P attenuates hepatocarcinogenesis via modulating glycolysis
Source: Oncogene. 2020 Jan 20;39(11):2408–23. doi: 10.1038/s41388-020-1158-y (PMC7067692; doi:10.1038/s41388-020-1158-y)
Supplement: Supplementary file 4 — Supplementary Table 3 [file 41388_2020_1158_MOESM4_ESM.pdf]

Table S3. The list of WFDC21P-interacting proteins identified by mass spectrometry.

	Accession	Name	Species
1	sp|P04264|K2C1_HUMAN	Keratin, type II cytoskeletal 1 OS=Homo sapiens GN=KRT1 PE=1 SV=6	HUMAN
2	sp|P07355|ANXA2_HUMAN	Annexin A2 OS=Homo sapiens GN=ANXA2 PE=1 SV=2	HUMAN
3	sp|P13645|K1C10_HUMAN	Keratin, type I cytoskeletal 10 OS=Homo sapiens GN=KRT10 PE=1 SV=6	HUMAN
4	sp|P22626|ROA2_HUMAN	Heterogeneous nuclear ribonucleoproteins A2/B1 OS=Homo sapiens GN=HNRNPA2B1 PE=1 SV=2	HUMAN
5	sp|P14923|PLAK_HUMAN	Junction plakoglobin OS=Homo sapiens GN=JUP PE=1 SV=3	HUMAN
6	sp|P15924|DESP_HUMAN	Desmoplakin OS=Homo sapiens GN=DSP PE=1 SV=3	HUMAN
7	sp|P09651|ROA1_HUMAN	Heterogeneous nuclear ribonucleoprotein A1 OS=Homo sapiens GN=HNRNPA1 PE=1 SV=5	HUMAN
8	sp|O43790|KRT86_HUMAN	Keratin, type II cuticular Hb6 OS=Homo sapiens GN=KRT86 PE=1 SV=1	HUMAN
9	sp|P35908|K22E_HUMAN	Keratin, type II cytoskeletal 2 epidermal OS=Homo sapiens GN=KRT2 PE=1 SV=2	HUMAN
10	sp|P62873|GBB1_HUMAN	Guanine nucleotide-binding protein G(I)/G(S)/G(T) subunit beta-1 OS=Homo sapiens GN=GNB1 PE=1 SV=3	HUMAN
11	sp|Q92764|KRT35_HUMAN	Keratin, type I cuticular Ha5 OS=Homo sapiens GN=KRT35 PE=2 SV=5	HUMAN
12	sp|P78386|KRT85_HUMAN	Keratin, type II cuticular Hb5 OS=Homo sapiens GN=KRT85 PE=1 SV=1	HUMAN
13	sp|P63261|ACTG_HUMAN	Actin, cytoplasmic 2 OS=Homo sapiens GN=ACTG1 PE=1 SV=1	HUMAN
14	sp|P60174|TPIS_HUMAN	Triosephosphate isomerase OS=Homo sapiens GN=TPI1 PE=1 SV=3	HUMAN
15	sp|P52907|CAZA1_HUMAN	F-actin-capping protein subunit alpha-1 OS=Homo sapiens GN=CAPZA1 PE=1 SV=3	HUMAN
16	sp|P68363|TBA1B_HUMAN	Tubulin alpha-1B chain OS=Homo sapiens GN=TUBA1B PE=1 SV=1	HUMAN
17	sp|P35527|K1C9_HUMAN	Keratin, type I cytoskeletal 9 OS=Homo sapiens GN=KRT9 PE=1 SV=3	HUMAN
18	sp|P62879|GBB2_HUMAN	Guanine nucleotide-binding protein G(I)/G(S)/G(T) subunit beta-2 OS=Homo sapiens GN=GNB2 PE=1 SV=3	HUMAN
19	sp|P11142|HSP7C_HUMAN	Heat shock cognate 71 kDa protein OS=Homo sapiens GN=HSPA8 PE=1 SV=1	HUMAN
20	sp|Q13835|PKP1_HUMAN	Plakophilin-1 OS=Homo sapiens GN=PKP1 PE=1 SV=2	HUMAN
21	sp|P62805|H4_HUMAN	Histone H4 OS=Homo sapiens GN=HIST1H4A PE=1 SV=2	HUMAN
22	sp|Q13885|TBB2A_HUMAN	Tubulin beta-2A chain OS=Homo sapiens GN=TUBB2A PE=1 SV=1	HUMAN
23	sp|P02533|K1C14_HUMAN	Keratin, type I cytoskeletal 14 OS=Homo sapiens GN=KRT14 PE=1 SV=4	HUMAN
24	sp|Q15323|K1H1_HUMAN	Keratin, type I cuticular Ha1 OS=Homo sapiens GN=KRT31 PE=2 SV=3	HUMAN
25	sp|P13647|K2C5_HUMAN	Keratin, type II cytoskeletal 5 OS=Homo sapiens GN=KRT5 PE=1 SV=3	HUMAN
26	sp|Q14525|KT33B_HUMAN	Keratin, type I cuticular Ha3-II OS=Homo sapiens GN=KRT33B PE=2 SV=3	HUMAN
27	sp|P31947|1433S_HUMAN	14-3-3 protein sigma OS=Homo sapiens GN=SFN PE=1 SV=1	HUMAN
28	sp|P30041|PRDX6_HUMAN	Peroxiredoxin-6 OS=Homo sapiens GN=PRDX6 PE=1 SV=3	HUMAN
29	sp|P04406|G3P_HUMAN	Glyceraldehyde-3-phosphate dehydrogenase OS=Homo sapiens GN=GAPDH PE=1 SV=3	HUMAN
30	sp|Q8TF66|LRC15_HUMAN	Leucine-rich repeat-containing protein 15 OS=Homo sapiens GN=LRRC15 PE=1 SV=2	HUMAN
31	sp|P16403|H12_HUMAN	Histone H1.2 OS=Homo sapiens GN=HIST1H1C PE=1 SV=2	HUMAN
32	sp|P08107|HSP71_HUMAN	Heat shock 70 kDa protein 1A/1B OS=Homo sapiens GN=HSPA1A PE=1 SV=5	HUMAN
33	sp|P47755|CAZA2_HUMAN	F-actin-capping protein subunit alpha-2 OS=Homo sapiens GN=CAPZA2 PE=1 SV=3	HUMAN
34	sp|P11021|GRP78_HUMAN	78 kDa glucose-regulated protein OS=Homo sapiens GN=HSPA5 PE=1 SV=2	HUMAN
35	sp|P05787|K2C8_HUMAN	Keratin, type II cytoskeletal 8 OS=Homo sapiens GN=KRT8 PE=1 SV=7	HUMAN

36	sp|Q5VTE0|EF1A3_HUMAN	Putative elongation factor 1-alpha-like 3 OS=Homo sapiens GN=EEF1A1P5 PE=5 SV=1	HUMAN
37	sp|Q99880|H2B1L_HUMAN	Histone H2B type 1-L OS=Homo sapiens GN=HIST1H2BL PE=1 SV=3	HUMAN
38	sp|P07195|LDHB_HUMAN	L-lactate dehydrogenase B chain OS=Homo sapiens GN=LDHB PE=1 SV=2	HUMAN
39	sp|Q71DI3|H32_HUMAN	Histone H3.2 OS=Homo sapiens GN=HIST2H3A PE=1 SV=3	HUMAN
40	sp|P04792|HSPB1_HUMAN	Heat shock protein beta-1 OS=Homo sapiens GN=HSPB1 PE=1 SV=2	HUMAN
41	sp|Q9NSB4|KRT82_HUMAN	Keratin, type II cuticular Hb2 OS=Homo sapiens GN=KRT82 PE=1 SV=3	HUMAN
42	sp|P31942|HNRH3_HUMAN	Heterogeneous nuclear ribonucleoprotein H3 OS=Homo sapiens GN=HNRNPH3 PE=1 SV=2	HUMAN
43	sp|P63104|1433Z_HUMAN	14-3-3 protein zeta/delta OS=Homo sapiens GN=YWHAZ PE=1 SV=1	HUMAN
44	sp|P13987|CD59_HUMAN	CD59 glycoprotein OS=Homo sapiens GN=CD59 PE=1 SV=1	HUMAN
45	sp|P68371|TBB4B_HUMAN	Tubulin beta-4B chain OS=Homo sapiens GN=TUBB4B PE=1 SV=1	HUMAN
46	sp|P60900|PSA6_HUMAN	Proteasome subunit alpha type-6 OS=Homo sapiens GN=PSMA6 PE=1 SV=1	HUMAN
47	sp|P62906|RL10A_HUMAN	60S ribosomal protein L10a OS=Homo sapiens GN=RPL10A PE=1 SV=2	HUMAN
48	sp|Q9BTM1|H2AJ_HUMAN	Histone H2A.J OS=Homo sapiens GN=H2AFJ PE=1 SV=1	HUMAN
49	sp|P39687|AN32A_HUMAN	Acidic leucine-rich nuclear phosphoprotein 32 family member A OS=Homo sapiens GN=ANP32A PE=1 SV=1	HUMAN
50	sp|P62136|PP1A_HUMAN	Serine/threonine-protein phosphatase PP1-alpha catalytic subunit OS=Homo sapiens GN=PPP1CA PE=1 SV=	HUMAN
51	sp|P46777|RL5_HUMAN	60S ribosomal protein L5 OS=Homo sapiens GN=RPL5 PE=1 SV=3	HUMAN
52	sp|Q9Y446|PKP3_HUMAN	Plakophilin-3 OS=Homo sapiens GN=PKP3 PE=1 SV=1	HUMAN
53	sp|P62937|PPIA_HUMAN	Peptidyl-prolyl cis-trans isomerase A OS=Homo sapiens GN=PPIA PE=1 SV=2	HUMAN
54	sp|P62241|RS8_HUMAN	40S ribosomal protein S8 OS=Homo sapiens GN=RPS8 PE=1 SV=2	HUMAN
55	sp|P62987|RL40_HUMAN	Ubiquitin-60S ribosomal protein L40 OS=Homo sapiens GN=UBA52 PE=1 SV=2	HUMAN
56	sp|P05187|PPB1_HUMAN	Alkaline phosphatase, placental type OS=Homo sapiens GN=ALPP PE=1 SV=2	HUMAN
57	sp|Q86SJ6|DSG4_HUMAN	Desmoglein-4 OS=Homo sapiens GN=DSG4 PE=1 SV=1	HUMAN
58	sp|Q13162|PRDX4_HUMAN	Peroxiredoxin-4 OS=Homo sapiens GN=PRDX4 PE=1 SV=1	HUMAN
59	sp|Q9NP59|S40A1_HUMAN	Solute carrier family 40 member 1 OS=Homo sapiens GN=SLC40A1 PE=1 SV=1	HUMAN
60	sp|Q13151|ROA0_HUMAN	Heterogeneous nuclear ribonucleoprotein A0 OS=Homo sapiens GN=HNRNPA0 PE=1 SV=1	HUMAN
61	sp|P02768|ALBU_HUMAN	Serum albumin OS=Homo sapiens GN=ALB PE=1 SV=2	HUMAN
62	sp|Q5VU13|VSIG8_HUMAN	V-set and immunoglobulin domain-containing protein 8 OS=Homo sapiens GN=VSIG8 PE=1 SV=1	HUMAN
63	sp|P27348|1433T_HUMAN	14-3-3 protein theta OS=Homo sapiens GN=YWHAQ PE=1 SV=1	HUMAN
64	sp|P31946|1433B_HUMAN	14-3-3 protein beta/alpha OS=Homo sapiens GN=YWHAB PE=1 SV=3	HUMAN
65	sp|P62258|1433E_HUMAN	14-3-3 protein epsilon OS=Homo sapiens GN=YWHAE PE=1 SV=1	HUMAN
66	sp|O76013|KRT36_HUMAN	Keratin, type I cuticular Ha6 OS=Homo sapiens GN=KRT36 PE=1 SV=1	HUMAN
67	sp|P06576|ATPB_HUMAN	ATP synthase subunit beta, mitochondrial OS=Homo sapiens GN=ATP5B PE=1 SV=3	HUMAN
68	sp|P08865|RSSA_HUMAN	40S ribosomal protein SA OS=Homo sapiens GN=RPSA PE=1 SV=4	HUMAN
69	sp|Q13228|SBP1_HUMAN	Selenium-binding protein 1 OS=Homo sapiens GN=SELENBP1 PE=1 SV=2	HUMAN
70	sp|P13639|EF2_HUMAN	Elongation factor 2 OS=Homo sapiens GN=EEF2 PE=1 SV=4	HUMAN
71	sp|P81605|DCD_HUMAN	Dermcidin OS=Homo sapiens GN=DCD PE=1 SV=2	HUMAN
72	sp|P62081|RS7_HUMAN	40S ribosomal protein S7 OS=Homo sapiens GN=RPS7 PE=1 SV=1	HUMAN
73	sp|Q8IUC1|KR111_HUMAN	Keratin-associated protein 11-1 OS=Homo sapiens GN=KRTAP11-1 PE=1 SV=1	HUMAN

74	sp	P51149	RAB7A_HUMAN	Ras-related protein Rab-7a OS=Homo sapiens GN=RAB7A PE=1 SV=1	HUMAN
75	sp	P48147	PPCE_HUMAN	Prolyl endopeptidase OS=Homo sapiens GN=PREP PE=1 SV=2	HUMAN
76	sp	P18124	RL7_HUMAN	60S ribosomal protein L7 OS=Homo sapiens GN=RPL7 PE=1 SV=1	HUMAN
77	sp	Q9BYU5	KRA21_HUMAN	Keratin-associated protein 2-1 OS=Homo sapiens GN=KRTAP2-1 PE=2 SV=2	HUMAN
78	sp	Q9BUL8	PDC10_HUMAN	Programmed cell death protein 10 OS=Homo sapiens GN=PDCD10 PE=1 SV=1	HUMAN
79	sp	Q15366	PCBP2_HUMAN	Poly(rC)-binding protein 2 OS=Homo sapiens GN=PCBP2 PE=1 SV=1	HUMAN
80	sp	P28838	AMPL_HUMAN	Cytosol aminopeptidase OS=Homo sapiens GN=LAP3 PE=1 SV=3	HUMAN
81	sp	P26641	EF1G_HUMAN	Elongation factor 1-gamma OS=Homo sapiens GN=EEF1G PE=1 SV=3	HUMAN
82	sp	P14618	KPYM_HUMAN	Pyruvate kinase PKM OS=Homo sapiens GN=PKM PE=1 SV=4	HUMAN
83	sp	P12004	PCNA_HUMAN	Proliferating cell nuclear antigen OS=Homo sapiens GN=PCNA PE=1 SV=1	HUMAN
84	sp	P07477	TRY1_HUMAN	Trypsin-1 OS=Homo sapiens GN=PRSS1 PE=1 SV=1	HUMAN
85	sp	Q10589	BST2_HUMAN	Bone marrow stromal antigen 2 OS=Homo sapiens GN=BST2 PE=1 SV=1	HUMAN
86	sp	Q3LI64	KRA61_HUMAN	Keratin-associated protein 6-1 OS=Homo sapiens GN=KRTAP6-1 PE=2 SV=1	HUMAN
87	sp	P37837	TALDO_HUMAN	Transaldolase OS=Homo sapiens GN=TALDO1 PE=1 SV=2	HUMAN
88	sp	P23396	RS3_HUMAN	40S ribosomal protein S3 OS=Homo sapiens GN=RPS3 PE=1 SV=2	HUMAN
89	sp	P12273	PIP_HUMAN	Prolactin-inducible protein OS=Homo sapiens GN=PIP PE=1 SV=1	HUMAN
90	sp	P18669	PGAM1_HUMAN	Phosphoglycerate mutase 1 OS=Homo sapiens GN=PGAM1 PE=1 SV=2	HUMAN
91	sp	P40925	MDHC_HUMAN	Malate dehydrogenase, cytoplasmic OS=Homo sapiens GN=MDH1 PE=1 SV=4	HUMAN
92	sp	Q53RT3	APRV1_HUMAN	Retroviral-like aspartic protease 1 OS=Homo sapiens GN=ASPRV1 PE=1 SV=1	HUMAN
93	sp	P17931	LEG3_HUMAN	Galectin-3 OS=Homo sapiens GN=LGALS3 PE=1 SV=5	HUMAN
94	sp	P08238	HS90B_HUMAN	Heat shock protein HSP 90-beta OS=Homo sapiens GN=HSP90AB1 PE=1 SV=4	HUMAN
95	sp	P22392	NDKB_HUMAN	Nucleoside diphosphate kinase B OS=Homo sapiens GN=NME2 PE=1 SV=1	HUMAN
96	sp	P35030	TRY3_HUMAN	Trypsin-3 OS=Homo sapiens GN=PRSS3 PE=1 SV=2	HUMAN
97	sp	P21291	CSRP1_HUMAN	Cysteine and glycine-rich protein 1 OS=Homo sapiens GN=CSRP1 PE=1 SV=3	HUMAN
98	sp	Q9Y4J8	DTNA_HUMAN	Dystrobrevin alpha OS=Homo sapiens GN=DTNA PE=1 SV=2	HUMAN
99	sp	O75083	WDR1_HUMAN	WD repeat-containing protein 1 OS=Homo sapiens GN=WDR1 PE=1 SV=4	HUMAN
100	sp	P25311	ZA2G_HUMAN	Zinc-alpha-2-glycoprotein OS=Homo sapiens GN=AZGP1 PE=1 SV=2	HUMAN
101	sp	Q5FWF4	ZRAB3_HUMAI	DNA annealing helicase and endonuclease ZRANB3 OS=Homo sapiens GN=ZRANB3 PE=1 SV=2	HUMAN
102	sp	Q92688	AN32B_HUMAN	Acidic leucine-rich nuclear phosphoprotein 32 family member B OS=Homo sapiens GN=ANP32B PE=1 SV=1	HUMAN
103	sp	P00403	COX2_HUMAN	Cytochrome c oxidase subunit 2 OS=Homo sapiens GN=MT-CO2 PE=1 SV=1	HUMAN
104	sp	P07947	YES_HUMAN	Tyrosine-protein kinase Yes OS=Homo sapiens GN=YES1 PE=1 SV=3	HUMAN
